# Supplementary material for: Evolution of noisy learning in games
Source: Proc Natl Acad Sci U S A. 2026 May 12;123(20):e2529959123. doi: 10.1073/pnas.2529959123 (PMC13187736; doi:10.1073/pnas.2529959123)
Supplement: Supplementary file 1 — Appendix 01 (PDF) [file pnas.2529959123.sapp.pdf]

Supporting Information for

Evolution of noisy learning in games

Marta C. Couto, Fernando P. Santos and Christian Hilbe

Marta C. Couto.
E-mail: m.gomesdacunhacouto@uva.nl

This PDF file includes:

Figs. S1 to S17
Table S1
SI References

Contents

1 Detailed description of the baseline model 3
- A Introspection dynamics . . . 3
- B Adaptive dynamics . . . 4
  - B.1 Evolutionary stability . . . 4
  - B.2 Ecological equilibrium and polymorphic populations . . . 5
  - B.3 Canonical equation of adaptive dynamics . . . 6
  - B.4 Dynamics for small β . . . 6

2 Further results on the baseline model 6
- A Super-game for a wide range of 2×2 stage games . . . 6
- B Trait evolution plots and canonical equation . . . 7
- C Stationary distribution of introspection dynamics with asymmetric payoff sensitivities . . . 7
- D Evolution of average cooperation and payoffs . . . 7
- E Stage games with more than one singular point . . . 7
- F Evolutionary outcomes for a wider range of games . . . 8
- G Multiplayer games . . . 8
- H Repeated games . . . 9

3 Analysis of an alternative learning model: Experience-weighted attraction learning 10
- A A formal description of experience-weighted attraction learning . . . 10
- B Numerical implementation . . . 11
- C Simulation results . . . 11
- D A variant of introspection dynamics with synchronous updating . . . 12

4 Supporting Figures 15
- A Introspection dynamics . . . 15
- B Experience-weighted attraction learning . . . 23

The present document consists of three main sections. First, we provide some additional information on our baseline model. This model considers introspection dynamics as the (short-run) learning process, and adaptive dynamics as the long-run evolutionary process (**Section 1**). Thereafter, we present some additional findings and expand our discussion of results that we only briefly mention in the main text (**Section 2**). Finally, we consider an alternative family of learning processes, experience-weighted attraction learning (**Section 3**).

1. Detailed description of the baseline model

Here, we provide all the details necessary for the theoretical analysis presented in the main text. In **Section 1A** we describe how to obtain the stationary distributions for the short-run dynamics. **Section 1B** is dedicated to the long-run dynamics. There, we introduce some basic concepts of adaptive dynamics that we use in our analysis.

A. Introspection dynamics. In most of the main text, we focus on individuals who adopt new strategies based on introspection dynamics (1, 2). According to this process, in each time step one individual is chosen at random and given an opportunity to update its strategy. In particular, we provided an explicit formula for the stationary distribution over the game outcomes in the special case that players have the same payoff sensitivity, $\beta_1 = \beta_2 = \beta$ (see Eq. [4] in the main text). In the following, we derive that expression, and we discuss the case of $\beta_1 \neq \beta_2$.

Following Couto et al. (1), we represent the learning dynamics as a Markov chain. The states correspond to the four possible outcomes of the game – **CC**, **CD**, **DC**, and **DD**. The feasible payoffs are given by the matrix

$$\begin{array}{c|cc} & \mathbf{C} & \mathbf{D} \\ \hline \mathbf{C} & 1 & -B \\ \mathbf{D} & 1-A & 0 \end{array}\,. \qquad [1]$$

To formalize the Markov chain, we denote the probability that players move from state i to j in one time step by T_{ij}, where $i, j \in \{\mathbf{CC}, \mathbf{CD}, \mathbf{DC}, \mathbf{DD}\}$. Because each player is chosen with probability 1/2 to revise their strategy, and because a revising player switches with probability ϕ (as defined by Eq. [2]) of the main text), the transition matrix $T := (T_{ij})$ takes the form

$$\begin{pmatrix} 1-\frac{1}{2(1+e^{\beta_1 A})}-\frac{1}{2(1+e^{\beta_2 A})} & \frac{1}{2(1+e^{\beta_2 A})} & \frac{1}{2(1+e^{\beta_1 A})} & 0 \\ \frac{1}{2(1+e^{-\beta_2 A})} & 1-\frac{1}{2(1+e^{-\beta_2 A})}-\frac{1}{2(1+e^{-\beta_1 B})} & 0 & \frac{1}{2(1+e^{-\beta_1 B})} \\ \frac{1}{2(1+e^{-\beta_1 A})} & 0 & 1-\frac{1}{2(1+e^{-\beta_1 A})}-\frac{1}{2(1+e^{-\beta_2 B})} & \frac{1}{2(1+e^{-\beta_2 B})} \\ 0 & \frac{1}{2(1+e^{\beta_1 B})} & \frac{1}{2(1+e^{\beta_2 B})} & 1-\frac{1}{2(1+e^{\beta_1 B})}-\frac{1}{2(1+e^{\beta_2 B})} \end{pmatrix}. \qquad [2]$$

Interestingly, the transition matrix [2] corresponds to that in page 6 of Ref.(1) after the transformation $\beta A \to \beta_1 A$, $\beta A' \to \beta_2 A$, $\beta B \to \beta_1 B$, and $\beta B' \to \beta_2 B$. This is so because the learning dynamics of our game is equivalent to that of an asymmetric game in which (otherwise symmetric) payoffs are scaled by each player's respective payoff sensitivity.

For any finite value of β_1 and β_2, the transition matrix [2] is primitive. Hence, by the theorem of Perron-Frobenius, the distribution of reached game outcomes approaches a unique stationary distribution

$$\mathbf{u}(A, B, \beta_1, \beta_2) = (u_{CC}, u_{CD}, u_{DC}, u_{DD})$$

that depends on the game payoffs and the players' payoff sensitivities. This distribution can be computed as the unique solution of the eigenvector problem

$$\begin{aligned} \mathbf{u} &= \mathbf{u}T \\ \mathbf{u}\,\mathbf{e}^\top &= 1. \end{aligned} \qquad [3]$$

Here, $\mathbf{e}$ denotes the 4-dimensional row-vector where each entry is equal to 1. The superscript $\top$ indicates transposition. Hence, the second equation is the usual normalization for a probability vector (requiring that the sum of all entries $\mathbf{u}$ is equal to 1). For 2-strategy games, we can obtain simple analytical expressions of the stationary distribution. For the special case $\beta_1 = \beta_2 = \beta$, the solution of [3] is

$$\mathbf{u}(A, B, \beta, \beta) = \frac{1}{2 + e^{A\beta} + e^{B\beta}}(e^{A\beta}, 1, 1, e^{B\beta}), \qquad [4]$$

as provided in the main text. For the asymmetric case $\beta_1 \neq \beta_2$, one can derive the following solution,

$$\begin{aligned}\mathbf{u}(A,B,\beta_1,\beta_2) = \frac{1}{f}(&e^{(A+B)\beta_1} + e^{(A+B)\beta_2} + 2e^{A(\beta_1+\beta_2)} + 2e^{A(\beta_1+\beta_2)+B\beta_1} + 2e^{A(\beta_1+\beta_2)+B\beta_2},\\ &2e^{A\beta_1} + 2e^{B\beta_2} + e^{(A+B)\beta_1} + e^{(A+B)\beta_2} + 2e^{A\beta_1+B\beta_2},\\ &2e^{B\beta_1} + 2e^{A\beta_2} + e^{(A+B)\beta_1} + e^{(A+B)\beta_2} + 2e^{A\beta_2+B\beta_1},\\ &e^{(A+B)\beta_1} + e^{(A+B)\beta_2} + 2e^{B(\beta_1+\beta_2)} + 2e^{A\beta_1+B(\beta_1+\beta_2)} + 2e^{A\beta_2+B(\beta_1+\beta_2)}).\end{aligned} \tag{5}$$

Here, f is a normalization factor,

$$\begin{aligned}f = 2\Big(&e^{A\beta_1} + e^{A\beta_2} + e^{B\beta_1} + e^{B\beta_2} + e^{A(\beta_1+\beta_2)} + e^{B(\beta_1+\beta_2)} + 2e^{(A+B)\beta_1} + 2e^{(A+B)\beta_2} +\\ &+ e^{A\beta_1+B\beta_2} + e^{A\beta_2+B\beta_1} + e^{A\beta_1+B(\beta_1+\beta_2)} + e^{A\beta_2+B(\beta_1+\beta_2)} + e^{A(\beta_1+\beta_2)+B\beta_1} + e^{A(\beta_1+\beta_2)+B\beta_2}\Big).\end{aligned} \tag{6}$$

Based on this stationary distribution, we can also derive the expected payoff of a player with payoff sensitivity $\beta = \beta_1$ against a player with $\beta = \beta_2$. It is given by

$$\Pi_{\beta_2}(\beta_1) = \mathbf{u}(A,B,\beta_1,\beta_2)\cdot(1,-B,1-A,0). \tag{7}$$

Using the expression [5], this payoff becomes

$$\begin{aligned}\Pi_{\beta_2}(\beta_1) =& \frac{1}{f}\Big(-2B(e^{A\beta_1} + e^{B\beta_2} + e^{A\beta_1+B\beta_2}) + 2(1-A)(e^{A\beta_2} + e^{B\beta_1} + e^{A\beta_2+B\beta_1})\\ &+ 2(e^{A(\beta_2+\beta_1)} + e^{A(\beta_2+\beta_1)+B\beta_2} + e^{A(\beta_2+\beta_1)+B\beta_1}) - (A+B-2)(e^{(A+B)\beta_1} + e^{(A+B)\beta_2})\Big).\end{aligned} \tag{8}$$

This equation is crucial for most of our results (as one example, it allows us to numerically produce Figure 4 of the main text).

B. Adaptive dynamics. In the following, we review some basic concepts of adaptive dynamics that we use in our subsequent analysis for the evolution of β. In the main text, we already define the key concepts of *invasion fitness*, *local fitness gradient* (or *selection gradient*), and *singular point* and respective evolutionary stability properties. Here, we extend some of these notions and introduce the *canonical equation* of adaptive dynamics. For that we follow Refs.(3–6).

As in the main text, we interpret β as a player's payoff sensitivity. In the following, we often need to refer to the payoff sensitivity of the resident population and to the payoff sensitivity of a mutant. To distinguish those two variables, it is convenient to use new variables: x for a resident's payoff sensitivity trait, and y for the mutant's trait.

B.1. Evolutionary stability. We first recall the definition of a mutant's *invasion fitness*. For a mutant with $\beta = y$ in a monomorphic resident population with trait $\beta = x$, this invasion fitness is given by

$$s_x(y) := \Pi_x(y) - \Pi_x(x), \tag{9}$$

Here, $\Pi_x(y)$ refers to a player's average payoff during the learning process, as given by Equation [8]. The invasion fitness is a central quantity as its properties determine the system's evolutionary fate. If $s_x(y)$ is positive, a mutant with trait y can invade a resident population with trait x. If $s_x(y)$ is negative, the respective mutant is expected to go extinct. The *selection gradient* is the derivative of the invasion fitness evaluated at the resident population x,

$$D(x) := \left[\frac{\partial s_x(y)}{\partial y}\right]_{y=x}. \tag{10}$$

Thus, the selection gradient determines the direction of evolutionary change. When $D(x)$ is positive (negative), mutants with a slightly higher (lower) trait value than x can replace the resident population. Therefore, we would expect the population's trait value to increase (decrease). A point x^* that satisfies $D(x^*) = 0$ is called evolutionary singular. To describe an evolutionary singular point's stability properties, let us define

$$\begin{aligned}a := a(x^*) &:= \left[\frac{\partial^2 s_x(y)}{\partial x^2}\right]_{y=x=x^*}\\ b := b(x^*) &:= \left[\frac{\partial^2 s_x(y)}{\partial y^2}\right]_{y=x=x^*}.\end{aligned} \tag{11}$$

Then a singular point's stability can be characterized as follows (3).

(i) If $b < 0$, no nearby mutant can invade. Therefore, the singular point is *evolutionarily stable.*

(ii) If $a - b < 0$, a population of a nearby trait can be invaded by mutants that are closer to x^*. Therefore, the singular point is *convergence-stable.*

(iii) If $a > 0$, the singular trait x^* can invade populations of a slightly different trait when initially rare itself.

(iv) If $a + b < 0$, all pairs of traits near the singular point can mutually invade each other. Therefore, there are nearby dimorphisms.

If (i) and (ii) are satisfied, x^* is called *continuously stable.* This means that the singular trait corresponds to an evolutionary endpoint. If (ii) but not (i) is satisfied, x^* is an *evolutionary branching point.*

B.2. Ecological equilibrium and polymorphic populations. Even though adaptive dynamics often describes transitions from one homogeneous population to another, the system can also reach a point where the mutant does not completely replace the resident. This occurs when the resident and mutant traits can mutually invade each other (for example, at a branching point). In that case, we would expect the evolution of a dimorphism, where the two traits coexist. In the following, we describe how to deal with those cases.

By the common assumption that mutations are rare, a resident population reaches an *ecological equilibrium* before a new mutant appears. Usually, that means a mutant either dies out or fixes in the population before a new mutant is introduced. However, if coexistences are allowed, there can be a stable ecological equilibrium among different types present in the population. The ecological equilibrium can also be regarded as the replicator equation equilibrium (6). At this equilibrium, all individuals have the same fitness (the same average payoff). If there are two coexisting types x_1 and x_2, we can calculate the ecological equilibrium point p, denoting the proportion of type x_1 in the population, by solving

$$p\,\Pi_{x_1}(x_1) + (1-p)\,\Pi_{x_2}(x_1) = p\,\Pi_{x_1}(x_2) + (1-p)\,\Pi_{x_2}(x_2). \qquad [12]$$

Here, the left-hand side refers to the average payoff of type x_1 and the right-hand side refers to the average payoff of type x_2, assuming a well-mixed population. The ecological stability is guaranteed because, by assumption, the two present types are able to mutually invade each other. Moreover, the uniqueness of the stable equilibrium point is guaranteed because the average payoffs depend linearly on the first type's frequency p.

Similarly, when there are three types, x_1, x_2 and x_3, we obtain the equilibrium point $(p_1, p_2, 1-p_1-p_2)$, where p_1 and p_2 respectively denote the proportion of types x_1 and x_2, by solving

$$\begin{aligned} p_1\,\Pi_{x_1}(x_1) + p_2\,\Pi_{x_2}(x_1) + (1-p_1-p_2)\,\Pi_{x_3}(x_1) &= p_1\,\Pi_{x_1}(x_2) + p_2\,\Pi_{x_2}(x_2) + (1-p_1-p_2)\,\Pi_{x_3}(x_2) \\ p_1\,\Pi_{x_1}(x_1) + p_2\,\Pi_{x_2}(x_1) + (1-p_1-p_2)\,\Pi_{x_3}(x_1) &= p_1\,\Pi_{x_1}(x_3) + p_2\,\Pi_{x_2}(x_3) + (1-p_1-p_2)\,\Pi_{x_3}(x_3). \end{aligned} \qquad [13]$$

Based on this ecological equilibrium, we can generalize the concept of invasion fitness to a polymorphic system. Suppose there are n different types or traits. Let the invasion fitness of a mutant y in a resident population with traits $x_1, \ldots, x_n$ at its ecological equilibrium be $s_{x_1,\ldots,x_n}(y)$. We define the direction of evolution of the x_i-trait by

$$D_i(x_1, \ldots, x_n) := \left[\frac{\partial s_{x_1,\ldots,x_n}(y)}{\partial y}\right]_{y=x_i}. \qquad [14]$$

For example, for $n = 2$ types, we get

$$D_i(x_1, x_2) := \left[\frac{\partial s_{x_1,x_2}(y)}{\partial y}\right]_{y=x_i}, \qquad [15]$$

for $i \in \{1, 2\}$. Here, the respective invasion fitness is

$$s_{x_1,x_2}(y) = \Pi_{x_1,x_2}(y) - \Pi_{x_1,x_2}(x_1), \qquad [16]$$

where

$$\begin{aligned} \Pi_{x_1,x_2}(y) &= p\,\Pi_{x_1}(y) + (1-p)\,\Pi_{x_2}(y) \\ \Pi_{x_1,x_2}(x_1) &= p\,\Pi_{x_1}(x_1) + (1-p)\,\Pi_{x_2}(x_1). \end{aligned} \qquad [17]$$

Recall that at the ecological equilibrium p we have $\Pi_{x_1,x_2}(x_1) = \Pi_{x_1,x_2}(x_2)$.

B.3. Canonical equation of adaptive dynamics. Since the selection gradients give the direction of trait evolution, we can use them to write a differential equation describing a deterministic approximation of the evolutionary dynamics. This equation is called the *canonical equation* of adaptive dynamics (5). For $n = 1$ type, it takes the form

$$\dot{x} = C_0\, D(x). \tag{18}$$

For $n = 2$ types,

$$\begin{aligned} \dot{x_1} &= C_1\, p\, D_1(x_1, x_2), \\ \dot{x_2} &= C_2\, (1-p)\, D_2(x_1, x_2). \end{aligned} \tag{19}$$

Finally, for $n = 3$ types,

$$\begin{aligned} \dot{x_1} &= C_1\, p_1\, D_1(x_1, x_2, x_3), \\ \dot{x_2} &= C_2\, p_2\, D_2(x_1, x_2, x_3), \\ \dot{x_3} &= C_3\, (1 - p_1 - p_2)\, D_3(x_1, x_2, x_3). \end{aligned} \tag{20}$$

Here, C_0, C_1, C_2, C_3 are constant coefficients (4, 5). We use the concept of mutual invasibility and the higher-dimensional canonical equations in **Fig. S2**, where we explore the dynamics of a stag-hunt game after evolutionary branching has occurred. We provide further details in the respective section.

B.4. Dynamics for small β. Given the non-trivial form of the payoff equation [8], it can be difficult to analytically infer the sign of the selection gradient at a given position. However, such an inference becomes possible when we assume that the relevant payoff sensitivities are small. In that case, we can approximate the payoff equation [8] by the constant and the linear terms of the respective Taylor expansion,

$$\Pi_{\beta_2}(\beta_1) \approx \frac{1}{4}\Big(2 - A - B\Big) + \frac{1}{32}\Big(3A^2 - 2AB + 3B^2\Big)\cdot \beta_1 + \frac{1}{32}\Big(-A^2 - B^2 - 4B + 4A + 6AB\Big)\cdot \beta_2 \tag{21}$$

The respective approximation for the invasion fitness [9] becomes

$$s_x(y) = \Pi_x(y) - \Pi_x(x) \approx \frac{1}{32}\Big(-3A^2 + 2AB - 3B^2\Big)\cdot x + \frac{1}{32}\Big(3A^2 - 2AB + 3B^2\Big)\cdot y \tag{22}$$

This expression allows us to easily compute the selection gradient [10] in the origin, for residents with payoff sensitivity $x = 0$,

$$D(0) = \left[\frac{\partial s_x(y)}{\partial y}\right]_{y=x=0} \approx \frac{1}{32}\Big(3A^2 - 2AB + 3B^2\Big) = \frac{1}{32}\Big(2(A^2 + B^2) + (A - B)^2\Big). \tag{23}$$

We note that this expression satisfies $D(0) \geq 0$, with equality if and only if $A = B = 0$. That is, initially, for $x = 0$, the selection gradient is always positive, except for the degenerate case when the payoff matrix [1] takes the form

$$\begin{array}{c|cc} & \textbf{C} & \textbf{D} \\ \hline \textbf{C} & 1 & 0 \\ \textbf{D} & 1 & 0 \end{array}\,. \tag{24}$$

In this degenerate case, a player's payoff is independent of their own strategy. Thus, all strategies (and hence all payoff sensitivities) fare equally well.

2. Further results on the baseline model

A. Super-game for a wide range of 2×2 stage games. We refer to the interaction among players with different payoff sensitivities as a *super-game*. Specifically, in a super-game, strategies are defined by a player's payoff sensitivity and the respective payoffs result from the introspection dynamics stationary distribution (see **Fig. 2D, H** of the main text). In **Fig. 2D**, the high β dominates the low β (in this case, $\beta = 5$ dominates $\beta = 2$). The example in **Fig. 2H** depicts a case where the low β value is a best response to the high β value and vice versa. Each of these two examples corresponds to one particular stage game and one pair of payoff sensitivities. We can do the same exercise for any 2×2 stage game, and for any pair of β values. In **Fig. S1**, we show the resulting super-game for three different pairs of payoff sensitivities and a wide range of stage games. There are regions for all possible cases: the higher β dominating the lower one, the lower β dominating the higher one, coordination games where players prefer to have the same payoff sensitivity, and anti-coordination games, where players prefer to have the opposite payoff sensitivity than their opponent.

B. Trait evolution plots and canonical equation. In the main text, we have seen that stag-hunt games can lead to evolutionary branching. Here, we would like to further explore the subsequent dynamics after branching has occurred. In **Fig. S2**, we show in more detail the branching case presented in the main text. First, we show again the pairwise invasibility plot (**Fig. S2A**), where we can see the singular branching point. Then, a *trait evolution plot* (3, 5) (**Fig. S2B**) highlights which trait types can coexist. The gray-shaded area corresponds to trait pairs x_1 and x_2 that can mutually invade each other, such that $s_{x_1}(x_2) > 0$ and $s_{x_2}(x_1) > 0$. Therefore, we can obtain a trait evolution plot by overlapping the original pairwise invasibility plot with its mirror image (obtained by inverting the pairwise invasibility plot with respect to the main diagonal). We can focus on the half above the diagonal only, defining the low trait (branch) to be x_1 and the high trait to be x_2. The lower half of the plot corresponds to the symmetric case. The gradient $(D_1(x_1, x_2), D_2(x_1, x_2))$ (gray vector field overlapping the shaded area) shows the dynamics of the branches x_1 and x_2; the colored lines correspond to isoclines (where D_1 or D_2 vanish). In this case, the isoclines do not intersect – there is always change at least in one of the directions. In particular, the upper branch x_2 keeps growing (the gradient points upwards), while the bottom branch x_1 seems to stabilize somewhere between 1 and 2. We confirm that by solving the canonical equation numerically (**Fig. S2C**). This analysis also reveals that the subpopulation corresponding to the lower branch is larger, reaching an abundance of about 79% (**Fig. S2C**, bottom).

C. Stationary distribution of introspection dynamics with asymmetric payoff sensitivities. Analogously to **Fig. 2** in the main text, we can derive super-game payoff matrices for other stage games. In **Fig. S3**, we do this for three other games, the stag-hunt game (panels A–D), a weak prisoner's dilemma (E–H), and a weak harmony game (I–L). Here, 'weak' refers to games where the payoff component $|A|$ is small.

In the stag-hunt game, as we increase the payoff sensitivity β of both players (**Fig. S3A–B**), players increasingly learn to play **D** – because that is the risk-dominant strategy. Therefore, when both players have the same high payoff sensitivity (**Fig. S3B**), both of them get a low payoff close to zero. In this scenario, we observe that the super-game takes the form of an anti-coordination game (**Fig. S3D**). That is, when both players have a high payoff sensitivity, there is an incentive to deviate towards lower payoff sensitivity. A player with lower payoff sensitivity would play **C** for a substantial amount of time. The player with higher payoff sensitivity would then best-respond, which in turn makes them play **C** a considerable amount of time, too. This translates into higher payoffs for both players, providing an intuition for why branching occurs.

For a weak prisoner's dilemma with small $|A|$, the gain in deviating from mutual cooperation is small compared to the mutual cooperation reward. Therefore, by increasing one's β (player 1 in **Fig. S3E to G**), the small gain from increasing the abundance of state **DC** is not enough to compensate for the increase in mutual defection (at the cost of decreasing mutual cooperation). On the other hand, lowering one's β (player 2 in **Fig. S3F to G**), and hence cooperating more, induces the co-player to cooperate slightly more, too, since there is less risk of being the sucker. That yields an increase in both players' average payoff. This shows that even in a prisoner's dilemma, the strictest kind of social dilemma, we can see the emergence of an ESS with finite payoff sensitivity.

Finally, we present an example of a weak harmony game. Similar to the reasoning in the other cases, we see from **Fig. S3I–K** that players spend most of the time playing mutual cooperation (the Nash equilibrium), and more so for higher β ($\beta_1 = \beta_2 = 15$). For the asymmetric case, however, we see that the player with larger payoff sensitivity cooperates more than the other. At the same time, given the more sensitive player cooperates, the difference between playing **C** and **D** to the low β player is negligible (0.05). Therefore, the low β individual plays **D** a significant amount of time. This, in turn incentivizes the high β player to cooperate even more often (because now the payoff difference between cooperation and defection is 0.5, rather than 0.05). As a result, the super-game takes the form of a prisoner's dilemma, **Fig. S3L**. Collectively, both players reach better decisions when they have a larger payoff-sensitivity. However, individually the player with lower sensitivity is more often in a high-payoff state.

D. Evolution of average cooperation and payoffs. In **Fig. S4** we show how the players' average cooperation rates and their average payoffs change according to the long-run dynamics, along with the trait β. For that, we assume all individuals in the population interact with each other. From time to time during a simulation, we then calculate the average cooperation rate and the payoff of an individual against every other. The average payoff is computed using Eq. (7). The average cooperation by a player with β_1 in an interaction with a player with β_2 is given by $u_{\mathbf{CC}}(A, B, \beta_1, \beta_2) + u_{\mathbf{CD}}(A, B, \beta_1, \beta_2)$. We then take the mean over all individuals in the population. Individuals start by cooperating half of the time since the initial population has $\beta = 0$. With increasing β, cooperation drops. Only in the stag-hunt game, cooperation rates eventually increase; here we observe that after the branching point, both subpopulations become more cooperative with time (see inset in **Fig. S4C**).

E. Stage games with more than one singular point. In **Fig. 4** of the main text, we study the adaptive dynamics of different stage games by varying the payoff parameters A and B. For each game, we report the lowest singular point

(the one which is reached from an initial population with payoff sensitivity $\beta=0$). Interestingly, however, some of those cases actually exhibit more than one evolutionary singular point – that is, the selection gradient [10] has more than one root. In **Fig. S5A-C**, we show three pairwise invasibility plots of such cases, namely a prisoner's dilemma, a snowdrift game, and a harmony game. For the three games, we see that the lowest singular point is around 4, and it is convergence-stable (left column of panels **A-C**). As we enlarge the range of the plot (middle and right columns), another singular point appears. This second point is divergent: above that point, the payoff sensitivity trait evolves towards ever higher values. In **Fig. S5D**, we show all games for which this happens. Those correspond exactly to the "outlier" regions of **Fig. 4** around the axis $A=0$. In these regions, the population evolves towards a finite singular point when starting from $\beta=0$, whereas it diverges towards ever-increasing payoff sensitivities when the initial β is sufficiently large.

F. Evolutionary outcomes for a wider range of games. In **Fig. S6**, we extend the range of one-shot games considered in **Fig. 4** of the main text. We obtain the same overall dynamics, except in the quadrant corresponding to stage games of the snowdrift type. In this quadrant, there is a white strip close to the diagonal for which there is no singular strategy. In the main text, we explained for a particular snowdrift game why we observe the evolution towards a finite value of β. The general mechanism is the following. When players with different β interact, the one with lower β is more prone to mistakes, that is, to deviate from one of the two Nash equilibria. Importantly, however, they are more likely to deviate from a Nash equilibrium when it is less costly to do so. The player with higher β, in turn, will on average react quicker to an unfavorable state by playing their best response. If this response makes them visit their less preferred equilibrium, then a player may have a disadvantage from having a higher β. In other words, the noisier player, by more frequently incurring short-term costs, pushes the more reactive player into an advantageous state for the former, ultimately leading to long-term gains. Overall, the conditions for the evolution of a finite value of β are:

1. For the row-player, **DC** is better than **CD** *and* deviations from **CD** are less costly than deviations from **DC**, *or*
2. For the row-player, **CD** is better than **DC** *and* deviations from **DC** are less costly than deviations from **CD**.

This translates into

$$\Big(-B < 1-A \ \wedge \ 0-(-B) > 1-(1-A)\Big) \vee \Big(-B > 1-A \ \wedge \ 0-(-B) < 1-(1-A)\Big). \qquad [25]$$

These conditions can be simplified to

$$\big(B > A\big) \ \vee \ \big(B < A-1\big). \qquad [26]$$

The expression in the first bracket refers to the upper left triangle in the snowdrift quadrant in **Fig. S6**. The expression in the second bracket refers to the bottom right triangle.

G. Multiplayer games. In the main text, we focus on simple matrix games among two players only. In the following, we briefly consider some special cases of multiplayer games. In particular, we analyze linear, discounted, and threshold public goods games. These games are considered multiplayer versions of the prisoner's dilemma, snowdrift, and stag-hunt games, respectively.

Again, we assume two strategies only. Players can either cooperate (**C**) by contributing to a common pool, or they defect (**D**) and contribute nothing. In the linear public goods game, each player decides whether to contribute or not a benefit r to the common pool at a cost of c. The sum of all benefits is equally divided among all N players. Thus, if a group has k cooperators, the payoffs of defectors and cooperators are, respectively,

$$\begin{aligned} \pi^{\mathbf{D}}(k) &= \frac{r}{N}k, \\ \pi^{\mathbf{C}}(k) &= \pi^{\mathbf{D}}(k) - c. \end{aligned} \qquad [27]$$

The discounted public goods game is similar to the linear public goods game. However, here each generated benefit gets discounted by a factor of $w < 1$, see Ref. (7). The payoffs are

$$\begin{aligned} \pi^{\mathbf{D}}(k) &= \frac{r}{N}(1 + w + w^2 + ... + w^{k-1}), \\ \pi^{\mathbf{C}}(k) &= \pi^{\mathbf{D}}(k) - c. \end{aligned} \qquad [28]$$

In the threshold public goods game, there is only a benefit to be redistributed if a minimum number of cooperators M is reached (8, 9). The payoffs are defined as

$$\pi^{\mathbf{D}}(k) = \frac{r}{N}\Theta(k-M),$$
$$\pi^{\mathbf{C}}(k) = \pi^{\mathbf{D}}(k) - c. \qquad [29]$$

Here, $\Theta(x)$ is the Heaviside function defined by $\Theta(x) = 1$ if $x \geq 0$ and $\Theta(x) = 0$ otherwise.

The expressions for the stationary distribution of introspection dynamics of multiplayer games (2) become much more cumbersome. As a result, an exact computation of the singular points of the respective adaptive dynamics is no longer possible. Instead, **Fig. S7** shows numerically generated pairwise invasibility plots for 3-player games. We find that the qualitative evolutionary outcomes are consistent with the respective 2-player game types in the main text.

H. Repeated games. Throughout our analysis, we have assumed that while players learn over the course of many interactions, they treat each interaction as a one-shot game. That is, we have neglected effects arising from direct reciprocity. This simplification has two reasons. First, it has a conceptual advantage: It allows us to introduce our framework in the most elementary setting, without having to describe an additional layer of decision-making in repeated games. Second, analytical results are easiest to achieve when there is only a finite number of strategies (in our case, we mostly considered simple 2×2 games). In contrast, when studying repeated games, scholars in evolutionary game theory often use the (uncountable) spaces of reactive strategies, memory-1 strategies, or finite-state automata (10–14). The dynamics among such strategies can be nontrivial even without considering the co-evolution of another parameter (15). This explains our focus on one-shot games.

Having said that, by restricting our attention to a finite subset of strategies, we can gain some insights into the co-evolution of direct reciprocity and payoff sensitivity already with our current methods. To illustrate this approach, we consider players who engage in a repeated donation game (16). That is, cooperating players pay a cost $c>0$ to give a benefit $b>c$ to the opponent. Defectors pay nothing and give nothing. The respective (one-shot) payoff matrix is

	C	D
C	$b-c$	$-c$
D	b	0

. [30]

We assume players engage in this game for infinitely many rounds, but future payoffs are discounted at a rate $\delta \in [0,1]$. Moreover, we assume that players choose among two strategies only, the well-known Tit-for-Tat (TFT) and Always defect ($ALLD$). As a result of these assumptions, we can derive a 2×2 matrix of the respective repeated game,

	TFT	$ALLD$
TFT	$b-c$	$-c(1-\delta)$
$ALLD$	$b(1-\delta)$	0

. [31]

For such a 2×2 game, our previous methods apply.

In **Fig. S8** we illustrate this approach with a specific numerical example, for $b=2$ and $c=1$. In that case, the payoff matrix further simplifies to

	TFT	$ALLD$
TFT	1	$-(1-\delta)$
$ALLD$	$2(1-\delta)$	0

. [32]

That is, the matrix is of the form [1], with $A=2\delta-1$ and $B=1-\delta$. The payoff matrix [32] represents an entire family of games, depending on the value of the parameter δ. In **Fig. S8**, this family is depicted as a black line (defined by $B=(-A+1)/2$). Interestingly, this line visits two quadrants of the game space: For $\delta<1/2$, the game satisfies the conditions of a prisoner's dilemma, whereas for $\delta>1/2$ it becomes a stag-hunt game. Even more, with respect to the evolution of payoff sensitivity, **Fig. S8** suggests that all three dynamical regimes are possible: β may increase indefinitely (e.g., for $\delta=0$ or $\delta=1$); it may converge towards a finite value (e.g., for $\delta=0.48$); or we may observe evolutionary branching (e.g., for $\delta=0.6$).

This result illustrates that already the repeated donation game with only two strategies can generate many of our previously described qualitative results. However, a more thorough analysis of our framework in the context of direct reciprocity would certainly be desirable. Respective studies could, for example, allow for more strategies and different payoff matrices. Moreover, the above exposition assumes a complete separation of time scales (between game dynamics, learning dynamics, and evolutionary dynamics). Future studies could weaken that assumption.

3. Analysis of an alternative learning model: Experience-weighted attraction learning

In our analysis so far we use introspection dynamics as the short-run learning dynamics of our model. To assess the generality of the respective results, we analyze an alternative family of learning models, *experience-weighted attraction* (EWA) learning (17–19). This family includes many standard learning rules as special cases, such as fictitious play and different variants of reinforcement learning. In contrast to introspection dynamics, however, there is in general no analytical formula for the average payoffs of two players who learn according to EWA. As a result, many results presented in the following will be based on simulations of the learning process.

A. A formal description of experience-weighted attraction learning. For the following account of EWA learning, we adapt the description of Dridi and Lehmann (20) to the case of 2-player games. The model assumes that each player i has a motivation $M_{i,t}(a)$ to play action a at time t. This motivation is updated as follows

$$M_{i,t+1}(a) = \frac{n_{i,t}}{n_{i,t+1}}\,\phi_{i,t}\,M_{i,t}(a) + \frac{1}{n_{i,t+1}}\left[\delta_i + (1-\delta_i)\mathbb{1}(a, a_{i,t})\right]\pi_i(a, a_{-i,t}) \qquad [33]$$

where

$$n_{i,t+1} = 1 + \rho_i n_{i,t} \qquad [34]$$

and where $\mathbb{1}(a, a_{i,t})$ is an indicator function,

$$\mathbb{1}(a, a_{i,t}) = \begin{cases} 1, & \text{if } a_{i,t} = a \\ 0, & \text{otherwise.} \end{cases} \qquad [35]$$

The interpretation of Eq. [33] is as follows. The first term refers to the weight of the previous motivation (at time t) when determining the current one (at time $t+1$). The parameter $\phi_{i,t} \geq 0$ relates to the player's memory, indicating how well player i remembers the previous motivation. The ratio $n_{i,t}/n_{i,t+1}$ reflects to which extent player i discounts the previous steps of play. In particular, if the parameter $\rho_i \in [0,1]$ satisfies $\rho_i = 1$ (and $n_{i,1} = 1$), then $n_{i,t} = t$. In this case, the player assigns the same weight to each previous motivation. If, however, $\rho_i = 0$, then $n_{i,t} = 1$ for all t, and the individual gives disproportionate weight to the current motivation.

The second term in Eq. [33] refers to the impact of the current outcome on the player's motivation. Here, $\pi_i(a, a_{-i,t})$ denotes player i's payoff, given the other player uses strategy $a_{-i,t}$. The central parameter in this second term is $\delta_i \in [0,1]$, which tunes the importance of the indicator function. When $\delta_i = 0$, the second term becomes $(1/n_{i,t+1})\mathbb{1}(a, a_{i,t})\pi_i(a, a_{-i,t})$. This term is non-zero only for $a = a_{i,t}$. That is, only the motivation for the action currently played is affected. In contrast, when $\delta_i = 1$, the term becomes $(1/n_{i,t+1})\pi_i(a, a_{-i,t})$. Hence, the player's motivation for each action a is updated, based on the payoff the player would have obtained had they played a at time t (even if they actually did not play it). Therefore, δ_i captures a player's ability to mentally simulate non-realized payoffs, or to think counterfactually – similar to what is assumed under introspection dynamics.

Motivations translate into how likely a player chooses each action. One widely adopted choice rule is the logit rule,

$$p_{i,t}(a) = \frac{\exp[\lambda_i M_{i,t}(a)]}{\sum_{k\in\mathcal{A}} \exp[\lambda_i M_{i,t}(k)]}. \qquad [36]$$

Here, the relevant learning parameter is λ_i. It represents an individual's sensitivity to motivations, errors in decision-making, or a propensity to explore the whole set of actions A (even those with low motivation). When $\lambda_i \to 0$, action a is chosen with probability $p_{i,t}(a) \to 1/m$ (where m is the number of available actions). In this case, choices are fully random, and individual i is a pure explorer. As $\lambda_i \to \infty$, the action with the highest motivation is chosen almost certainly. We refer to λ as a player's motivation sensitivity; it naturally corresponds to a player's payoff sensitivity β in the previous model of introspection dynamics. Thus, we identify the two parameters λ and β with each other in the following.

In analogy to our previous analysis of introspection dynamics, we allow λ_i to differ among individuals. All remaining parameters, $\phi_{i,t}$, ρ_i, and δ_i, are assumed to be the same for all players (so we drop their index i in the following). Depending on the particular choice of these parameters, we obtain different special cases of experience-weighted attraction learning. Out of the special cases presented in Table 1 of Dridi and Lehmann (20), we study those four which (i) allow for finite values of λ_i (necessary for a comparison with our setup) and (ii) use the logit function as probabilistic choice rule (also more directly comparable to introspection dynamics). The four models we consider are summarized in **Table S1**. For more details on the relation of these models (and others) to the previous literature, we refer to Dridi and Lehmann (20).

Table S1. Special cases of experience-weighted attraction learning with $\rho = 1$ and $\lambda_i > 0$.

	$\delta = 1$	$\delta = 0$
$\phi_t = 1$	Stochastic fictitious play (FP)	Exploratory reinforcement learning (ERL)
$\phi_t = 1 + \frac{1}{t}$	Payoff-informed learning (IL)	Pure reinforcement learning (PRL)

B. Numerical implementation. When we studied the impact of payoff sensitivity β on introspection dynamics, our analysis was facilitated by the fact that there is an analytical expression for the resulting stationary distribution, Eq. [5]. By using the respective formula, we could derive an explicit equation $\Pi_{\beta_2}(\beta_1)$ for the payoff of a learner with payoff sensitivity β_1 against an opponent with sensitivity β_2, see Eq. [8]. Conveniently, this payoff was independent of the players' actions in the very first round. Based on this payoff expression, we could fully characterize the resulting adaptive dynamics of β, for any given 2×2 stage game.

For the general form of experience-weighted attraction learning we are not aware of an analogous formula $\Pi_{\lambda_2}(\lambda_1)$ as a function of a player's motivation sensitivity. In the following, we thus rely on simulations to estimate the respective payoff. These simulations use the following setup:

(*i*) For two players with fixed sensitivities λ_1 and λ_2 we run `nIterations` many independent simulations of the learning process.

(*ii*) For each simulation we assume that players initially have a motivation of $M_{i,0}(a) = 0$ for each action $a \in \{C, D\}$. We also explored alternative scenarios where initial motivations are uniformly distributed in (0,1). The respective results differ quantitatively from the results reported below, but the main qualitative conclusions remain unchanged.

(*iii*) For each individual simulation run, we simulate the EWA learning process outlined in Section A for t running from 0 to `maxTime`. We define each player's payoff as their average payoff across the second half of the simulation run. This was done to minimize the impact of the initial rounds on the players' payoffs (the assumption of only considering the second half has a similar effect to increasing `maxTime`, but it has a lower computational cost).

(*iv*) By averaging the payoffs per simulation across all `nIterations` many simulations, we obtain an estimate for a player's expected payoff $\Pi_{\lambda_2}(\lambda_1)$.

Because payoffs are now simulated (instead of calculated), the respective procedure is computationally expensive. As a result, it is no longer feasible to simulate an entire population's adaptive dynamics (as in **Fig. 3A,C,E** in the main text). However, the above approach still allows us to estimate expected payoffs for any pair (λ_1, λ_2), and hence to numerically compute pairwise invasibility plots. In the following Section C, we present results for the four learning rules described in Table S1. The respective MATLAB code is provided online. For these simulations, we either use `maxTime` $= 200$ (when we illustrate some representative dynamics of motivations) or `maxTime` $= 2,000$ (when we estimate the players' expected payoffs). The respective payoff estimations are based on `nIterations` $= 10^5$ many simulations, unless noted otherwise. Moreover, in Section D, we compare these numerically derived plots with exact plots in a special case where analytical results are feasible. These latter results provide some support for the reliability of the above simulation algorithm.

C. Simulation results. As with introspection dynamics, we observe many cases in which larger motivation sensitivities are always favored. However, for each learning rule in **Table S1**, we also observe exceptions. One such exception is shown in **Fig. S9**. This figure considers two players who use pure reinforcement learning (PRL) to learn strategies in a snowdrift game with payoff parameters $A = -0.6$ and $B = -1$ (**Fig. S9A**). If we restrict players to either have a motivation sensitivity of $\lambda = 2$ or $\lambda = 5$, we make a similar observation as in previous snowdrift games. Players are most likely to find themselves in one of the two asymmetric Nash equilibria (C, D) and (D, C); yet when players have different motivation sensitivities, the one with lower λ spends more time in its preferred equilibrium (**Fig. S9B–D**). As a result, in the respective super-game, a low motivation sensitivity dominates the high one (**Fig. S9E**).

Interestingly, the respective simulated payoff matrices in **Fig. S9B–D** suggest that players also spend a substantial amount of time in the non-equilibrium state (C, C). To explore these cases, we show three typical learning dynamics that we observed across the different simulation runs, see **Fig. S9F**. In the first two, one player eventually shows a large motivation for cooperation whereas the other player shows a large motivation for defection (consistent with equilibrium play). In the last case, however, both players show a large motivation for cooperation. This case typically occurs when both players happen to cooperate early on in the beginning of the simulation run. Because the mutual cooperation payoff is positive, their motivation to cooperate further increases, which leads both players to be biased

towards cooperation. Even though both players also occasionally defect (because of their λ being finite), the larger (D,C)-payoff is not sufficient to outweight the regular positive reinforcement after obtaining the (C,C)-payoff.

As we perform similar simulations for all motivation sensitivities $\lambda \in \{0.0, 0.5, 1.0, \ldots, 10.0\}$, we obtain the pairwise invasibility plot shown in **Fig. S9G**. This numerically derived plot suggests the existence of a singular strategy at $\lambda \approx 1$. We end up with two conclusions: (*i*) Also for pure reinforcement learning, there are cases in which adaptive populations (starting from $\lambda = 0$) converge towards a state with finite motivation sensitivity. (*ii*) For the same stage game (with $A = -0.6$ and $B = -1$), introspection dynamics would have led to ever-increasing payoff sensitivities (see **Fig. 4**). Thus, the two learning rules differ in the payoff regions that lead to finite payoff/motivation sensitivities.

In a next step, we explore evolution in the same game, but now using a different learning rule, exploratory reinforcement learning (ERL), see **Fig. S10**. If we again restrict attention to players with two possible motivation sensitivities, $\lambda = 2$ or $\lambda = 5$, we again observe that in mixed groups, the player with low λ gets to spend more time in its preferred equilibrium. However, the resulting learning dynamics are slightly different to the ones described earlier (**Fig. S9**). Because the memory parameter ϕ_t is now smaller (1 instead of $1 + 1/t$), motivations converge towards smaller absolute values (**Fig. S10F**). As a result, cooperation decisions tend to be more noisy for given (finite) λ values (especially for players with the lower λ value). Overall, we only observe two main regimes (corresponding to noisy versions of the two pure equilibria), with the regime favoring the low-λ player arising disproportionately often. As a result, as we vary λ, we again observe the existence of a finite singular point (**Fig. S10G**).

The existence of finite singular points is not restricted to snowdrift games, nor does it require learning rules based on reinforcement learning. Instead, **Fig. S11** shows five different examples involving all learning rules in **Table S1** and different versions of stag-hunt or harmony games. In each case, we observe finite singular points, even though the specific mechanisms are different. In the depicted stag-hunt games, we often observe that mixed groups of players with different λ are more effective in coordinating on the payoff-dominant (C,C) equilibrium. In the depicted harmony games, a player with high λ is more likely to be swayed to cooperate when encountering a player with a low λ (who occasionally defects). In all depicted cases, the resulting pairwise invasibility plot suggests the existence of a finite evolutionarily stable state.

To obtain a more systematic picture of how pervasive these cases are, we systematically vary the two payoff parameters in discrete steps, $A, B \in \{-1.0, -0.6, -0.2, 0.2, 0.6, 1.0\}$. In each case, we numerically compute the resulting pairwise invasibility plot, for pure reinforcement learning (**Fig. S12**), exploratory reinforcement learning (**Fig. S13**), fictitious play (**Fig. S14**) and payoff-informed learning (**Fig. S15**). We observe the following patterns. (*i*) For all learning rules, one can find snowdrift and stag-hunt games such that there is a finite singular point, see the upper right and the lower left quadrants in the respective figures. (*ii*) In addition, reinforcement-learning based rules (but not the other two rules) seem to allow for a finite singular point in harmony games. (*iii*) We observe no finite singular point in any of the prisoner's dilemma cases.

However, when interpreting these graphs, several remarks are in order. First, for computational reasons we only plot the region $\lambda \in [0, 10]$. Hence, we may miss singular points outside this range. Second, again for computational reasons we only considered a rather coarse grid of payoff values A and B. We may observe further interesting cases as we choose different payoff values. Finally, since all pairwise invasibility plots are numerically generated, it can be difficult to determine whether a singular point (if it exists) gives rise to an evolutionarily stable rest point or to evolutionary branching. However, in our view the mere existence of these finite singular points (across all considered learning rules) remains remarkable.

D. A variant of introspection dynamics with synchronous updating. We note that the introspection dynamics model considered throughout most of the main text is not a special case of experience-weighted attraction learning. After all, introspection dynamics assumes that at any given time point at most one of the players gets to update its action (1, 2). However, within the traditional EWA framework, we can instantiate a learning rule similar to introspection dynamics by setting $\phi_t = 0$, $\rho = 0$ and $\delta = 1$ (this learning rule has not been considered specifically by Dridi and Lehmann, Ref. (20)). Since $\phi_t = 0$ and $\rho = 0$, the strategy update does not consider any information prior to the present time. Moreover, by setting $\delta = 1$ we allow for counterfactual updates. Note that with this parametrization, the motivation given by Eq. [33] reduces to $M_{i,t+1}(a) = \pi_i(a, a_{-i,t})$. We refer to this dynamics as 'introspection-like'. This dynamics is similar to our baseline model in the main text and in Section 1, except that at each time step of the learning process, both players might switch their strategies.

Again, because players are memory-less, we can describe introspection-like dynamics as a Markov chain, analogous to the approach in Section 1. We define the probability that players move from state i to j in one time step by T_{ij}^{sync},

where $i, j \in \{\mathbf{CC}, \mathbf{CD}, \mathbf{DC}, \mathbf{DD}\}$. The respective transition matrix $T^{sync} := (T_{ij}^{sync})$ takes the form

$$\begin{pmatrix} \frac{1}{(1+e^{-A\beta_1})(1+e^{-A\beta_2})} & \frac{1}{(1+e^{-A\beta_1})(1+e^{A\beta_2})} & \frac{1}{(1+e^{A\beta_1})(1+e^{-A\beta_2})} & \frac{1}{(1+e^{A\beta_1})(1+e^{A\beta_2})} \\ \frac{1}{(1+e^{B\beta_1})(1+e^{-A\beta_2})} & \frac{1}{(1+e^{B\beta_1})(1+e^{A\beta_2})} & \frac{1}{(1+e^{-B\beta_1})(1+e^{-A\beta_2})} & \frac{1}{(1+e^{-B\beta_1})(1+e^{A\beta_2})} \\ \frac{1}{(1+e^{-A\beta_1})(1+e^{B\beta_2})} & \frac{1}{(1+e^{-A\beta_1})(1+e^{-B\beta_2})} & \frac{1}{(1+e^{A\beta_1})(1+e^{B\beta_2})} & \frac{1}{(1+e^{A\beta_1})(1+e^{-B\beta_2})} \\ \frac{1}{(1+e^{B\beta_1})(1+e^{B\beta_2})} & \frac{1}{(1+e^{B\beta_1})(1+e^{-B\beta_2})} & \frac{1}{(1+e^{-B\beta_1})(1+e^{B\beta_2})} & \frac{1}{(1+e^{-B\beta_1})(1+e^{-B\beta_2})} \end{pmatrix}. \quad [37]$$

To obtain the stationary distribution $\mathbf{u^{sync}}(A, B, \beta_1, \beta_2)$ over the game outcomes, we again solve the eigenvector problem [3], yielding the unique solution

$$\begin{aligned} \mathbf{u^{sync}}(A,B,\beta_1,\beta_2) = \frac{1}{f}\Big(&\big(1+e^{A\beta_1}\big)\big(1+e^{A\beta_2}\big)\big(e^{A\beta_1}+e^{(A+B)\beta_1}+e^{A\beta_1+B\beta_2}+e^{B\beta_2}\big)\big(e^{A\beta_2}+e^{(A+B)\beta_2}+e^{A\beta_2+B\beta_1}+e^{B\beta_1}\big), \\ &\big(1+e^{B\beta_1}\big)\big(1+e^{A\beta_2}\big)\big(e^{A\beta_1}+e^{(A+B)\beta_1}+e^{A\beta_1+B\beta_2}+e^{B\beta_2}\big)\big(e^{A\beta_1}+e^{(A+B)\beta_2}+e^{A\beta_1+B\beta_2}+e^{B\beta_2}\big), \\ &\big(1+e^{A\beta_1}\big)\big(1+e^{B\beta_2}\big)\big(e^{A\beta_2}+e^{(A+B)\beta_1}+e^{A\beta_2+B\beta_1}+e^{B\beta_1}\big)\big(e^{A\beta_2}+e^{(A+B)\beta_2}+e^{A\beta_2+B\beta_1}+e^{B\beta_1}\big), \\ &\big(1+e^{B\beta_1}\big)\big(1+e^{B\beta_2}\big)\big(e^{A\beta_1}+e^{(A+B)\beta_2}+e^{A\beta_1+B\beta_2}+e^{B\beta_2}\big)\big(e^{A\beta_2}+e^{(A+B)\beta_1}+e^{A\beta_2+B\beta_1}+e^{B\beta_1}\big)\Big). \end{aligned} \quad [38]$$

Again, f is a normalization factor, given by

$$\begin{aligned} f = \Big(&e^{A\beta_1}+e^{B\beta_1}+2e^{(A+B)\beta_1}+e^{A\beta_2}+e^{B\beta_2}+2e^{(A+B)\beta_2}+e^{A(\beta_1+\beta_2)}+e^{B(\beta_1+\beta_2)}+e^{A\beta_2+B\beta_1} \\ &+e^{A\beta_1+B\beta_2}+e^{A(\beta_1+\beta_2)+B\beta_1}+e^{A(\beta_1+\beta_2)+B\beta_2}+e^{A\beta_1+B(\beta_1+\beta_2)}+e^{A\beta_2+B(\beta_1+\beta_2)}\Big)^2 \end{aligned}. \quad [39]$$

Based on this stationary distribution, we can again analytically compute a player's expected payoff $\Pi_{\beta_2}(\beta_1)$ according to Eq. [7], and the system's adaptive dynamics. The result is shown in **Fig. S17**, together with the numerically computed version based on the algorithm in Section B in **Fig. S16**. We make three observations: (*i*) The two independent methods, the simulations and the analytical approach, yield the same invasibility plots, which is reassuring. (*ii*) Again, the plots suggest the existence of finite singular points in the case of snowdrift games and stag-hunt games. (*iii*) Finally, we observe that on the main diagonal of **Fig. S16** and **Fig. S17**, for which $A=B$, all pairwise invasibility plots seem to be degenerated. Based on our analytical framework, we can make sense of this observation: For $A=B$, an (anti-)coordination game with equal gains from switching, the stationary distribution becomes simply $(\frac{1}{4}, \frac{1}{4}, \frac{1}{4}, \frac{1}{4})$, independent of the players' payoff sensitivity. This result may be surprising: it implies that players spend as much time off the two pure equilibria as they spend on the equilibrium outcomes. This suggests that synchronous updating can produce less sensible game outcomes (compared to the asynchronous introspection dynamics), as players may spend a long time simultaneously switchinig from one off-equilibrium outcome to another.

References

1. MC Couto, S Giaimo, C Hilbe, Introspection dynamics: A simple model of counterfactual learning in asymmetric games. *New J. Phys.* **24**, 63010 (2022).
2. MC Couto, S Pal, Introspection Dynamics in Asymmetric Multiplayer Games. *Dyn. Games Appl.* (2023).
3. SAH Geritz, E Kisdi, G Meszéna, JAJ Metz, Evolutionarily singular strategies and the adaptive growth and branching of the evolutionary tree. *Evol. Ecol. Res.* **12**, 35–57 (1998).
4. U Dieckmann, R Law, The dynamical theory of coevolution: a derivation from stochastic ecological processes. *J. Math. Biol.* **34**, 579–612 (1996).
5. Å Brännström, J Johansson, N von Festenberg, The Hitchhiker's guide to adaptive dynamics. *Games* **4**, 304–328 (2013).
6. M Doebeli, C Hauert, T Killingback, The evolutionary origin of cooperators and defectors. *Science* **306**, 859–62 (2004).
7. C Hauert, F Michor, MA Nowak, M Doebeli, Synergy and discounting of cooperation in social dilemmas. *J. Theor. Biol.* **239**, 195–202 (2006).

8. M Archetti, I Scheuring, Review: Evolution of cooperation in one-shot social dilemmas without assortment. *J. Theor. Biol.* **299**, 9–20 (2012).
9. JM Pacheco, FC Santos, MO Souza, B Skyrms, Evolutionary dynamics of collective action in n-person stag hunt dilemmas. *Proc. Royal Soc. B* **276**, 315–321 (2009).
10. LA Imhof, MA Nowak, Stochastic evolutionary dynamics of direct reciprocity. *Proc. Royal Soc. B* **277**, 463–468 (2010).
11. SK Baek, HC Jeong, C Hilbe, MA Nowak, Comparing reactive and memory-one strategies of direct reciprocity. *Sci. Reports* **6**, 25676 (2016).
12. J García, M van Veelen, No strategy can win in the repeated prisoner's dilemma: Linking game theory and computer simulations. *Front. Robotics AI* **5**, 102 (2018).
13. C Hilbe, K Chatterjee, MA Nowak, Partners and rivals in direct reciprocity. *Nat. Hum. Behav.* **2**, 469–477 (2018).
14. N Glynatsi, V Knight, A bibliometric study of research topics, collaboration and centrality in the field of the Iterated Prisoner's Dilemma. *Humanit. Soc. Sci. Commun.* **8**, 45 (2021).
15. MA Nowak, K Sigmund, Chaos and the evolution of cooperation. *Proc. Natl. Acad. Sci. USA* **90**, 5091–5094 (1993).
16. K Sigmund, *The Calculus of Selfishness.* (Princeton Univ. Press, Princeton, NJ), (2010).
17. CF Camerer, TH Ho, Experience-weighted attraction learning in normal form games. *Econometrica* **67**, 827–874 (1999).
18. C Camerer, *Behavioral Game Theory.* (Princeton Univ. Press, Princeton, NJ), (2003).
19. TH Ho, CF Camerer, JK Chong, Self-tuning experience weighted attraction learning in games. *J. Econ. Theory* **133**, 177–198 (2007).
20. S Dridi, L Lehmann, On learning dynamics underlying the evolution of learning rules. *Theor. Popul. Biol.* **91**, 20–36 (2014).

4. Supporting Figures

A. Introspection dynamics.

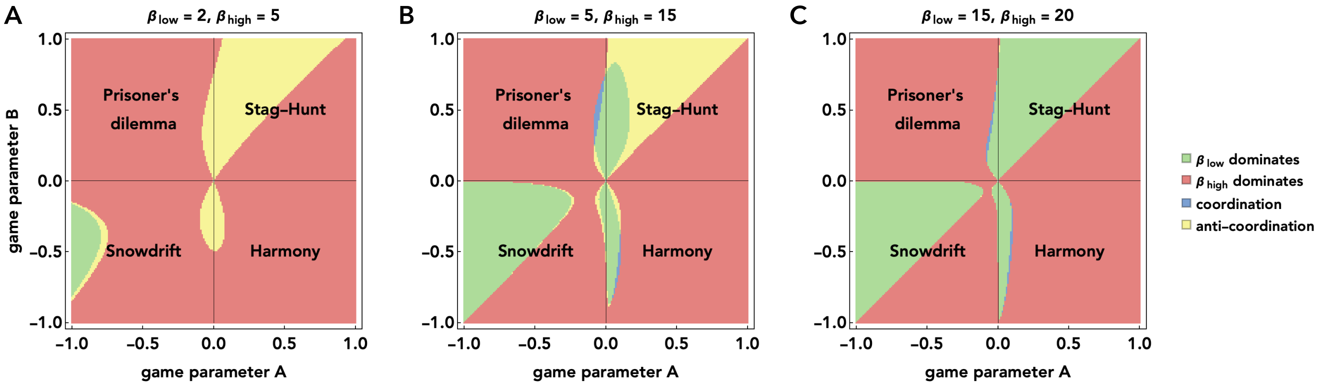

Fig. S1. Super-games for a wide range of 2×2 social dilemmas. We fix three pairs of payoff sensitivities: **A,** low sensitivities (2 and 5), **B,** intermediate sensitivities (5 and 15), and **C,** high sensitivities (15 and 20). Then we show, by varying the parameters A and B, for each stage game, the type of super-game that is created – low-β dominance, high-β dominance, coordination or anti-coordination.

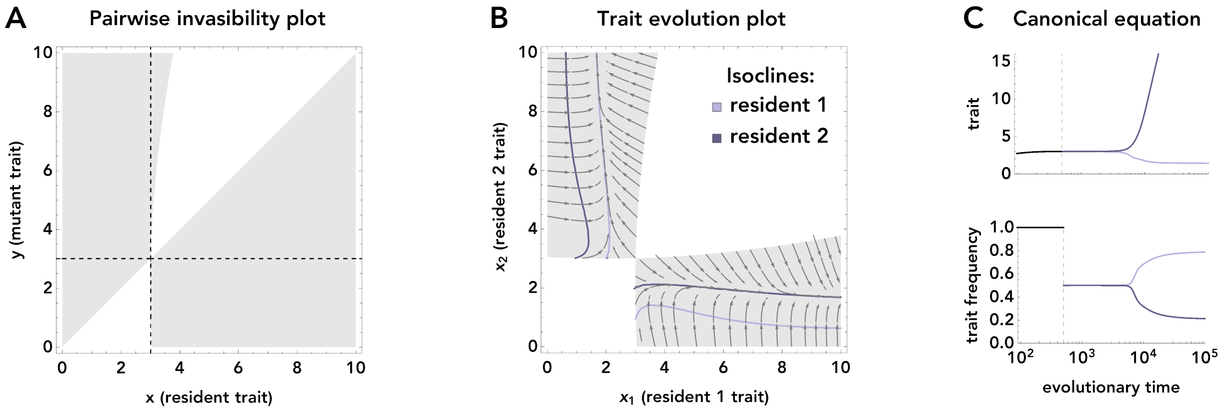

Fig. S2. Pairwise invasibility plots (A), trait evolution plots (B), and canonical equation (C) of the stag-hunt game with $A = 0.1, B = 0.8$**. A,** Shaded area shows $s_x(y) > 0$, as before. The dashed lines correspond to the singular points. **B,** Shaded area shows $s_{x_1}(x_2) > 0$ and $s_{x_2}(x_1) > 0$. The vector field corresponds to the gradient $(D_1(x_1, x_2), D_2(x_1, x_2))$, showing the direction of evolution. The colored lines correspond to isoclines, where D_1 or D_2 vanish, respectively. **C,** Canonical equation. We solve the canonical equations 18 and 19 numerically. The dashed line marks the branching point. The initial trait is 0, and $C_0 = 2, C_1 = C_2 = 5$. The trait frequencies are obtained by solving the ecological equilibrium condition Eq. 12.

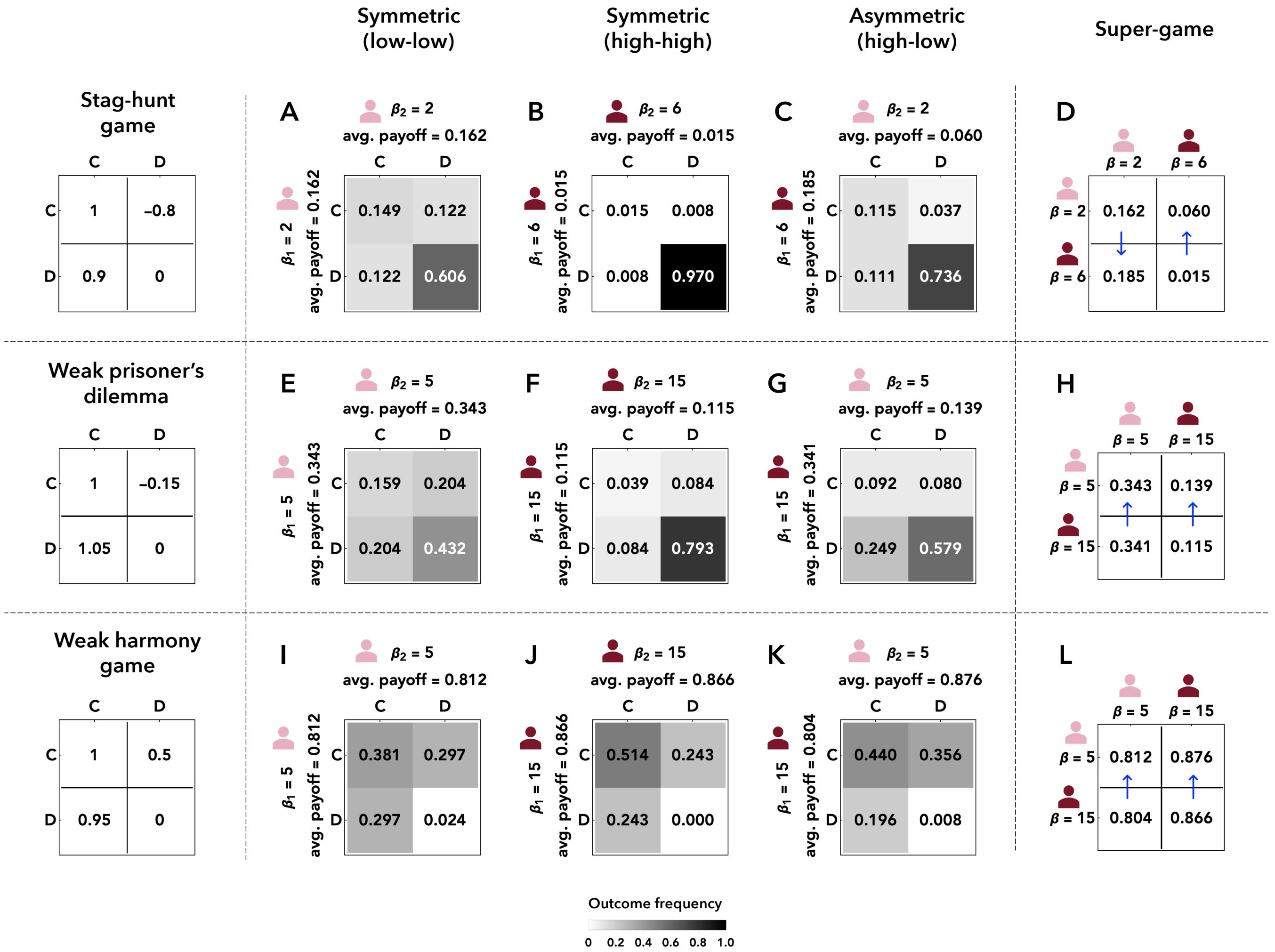

Fig. S3. Introspection dynamics among players with different payoff sensitivities. A–D, Stag-hunt game ($A = 0.1, B = 0.8$). **E–H,** Weak prisoner's dilemma ($A = -0.05, B = 0.15$). **I–L,** Weak harmony game ($A = 0.05, B = -0.5$). The stationary distribution is depicted by the black and white gradient – the darker the shading, the more frequent the game outcome. The numerical values of the stationary distribution are also shown. The average payoffs are calculated using Eq. 7. The blue arrows in the super-games indicate the preferred payoff sensitivity for a given opponent's sensitivity. For the stag-hunt game (**D**), a player prefers to have the high β when the opponent has the low β, and vice versa, whereas for the weak prisoner's dilemma (**H**) and harmony game (**L**), it's always preferable to have the low payoff sensitivity.

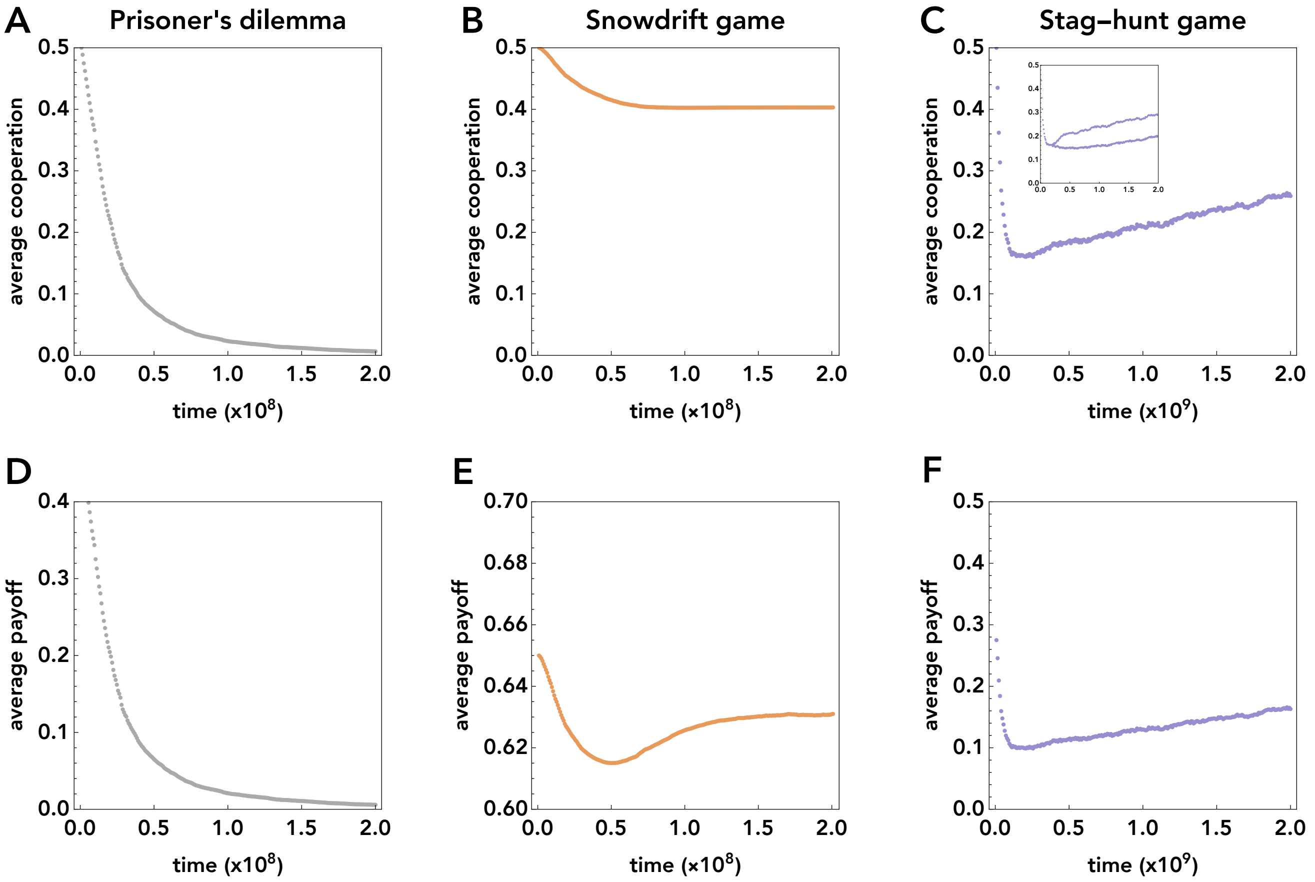

Fig. S4. Average cooperation (A–C) and average payoffs (D–E) over the whole population along evolutionary time. These are results from the same simulations shown in **Fig. 3** in the main text. The inset in panel **C** shows the average cooperation of each sub-population branch. The lower payoff sensitivity sub-population cooperates more: low β individuals cooperate more both when interacting among themselves compared to higher β individuals and when they interact with a high β individual. This can be confirmed in **Fig.** S3 **A–C**. However, the average payoffs in each branch are the same (**F**). This is because the two branches are in the "ecological equilibrium" (see **Eq.** 12). By construction, the fraction of individuals in each branch evolves to be such that all individuals in the population obtain the same average payoff (3).

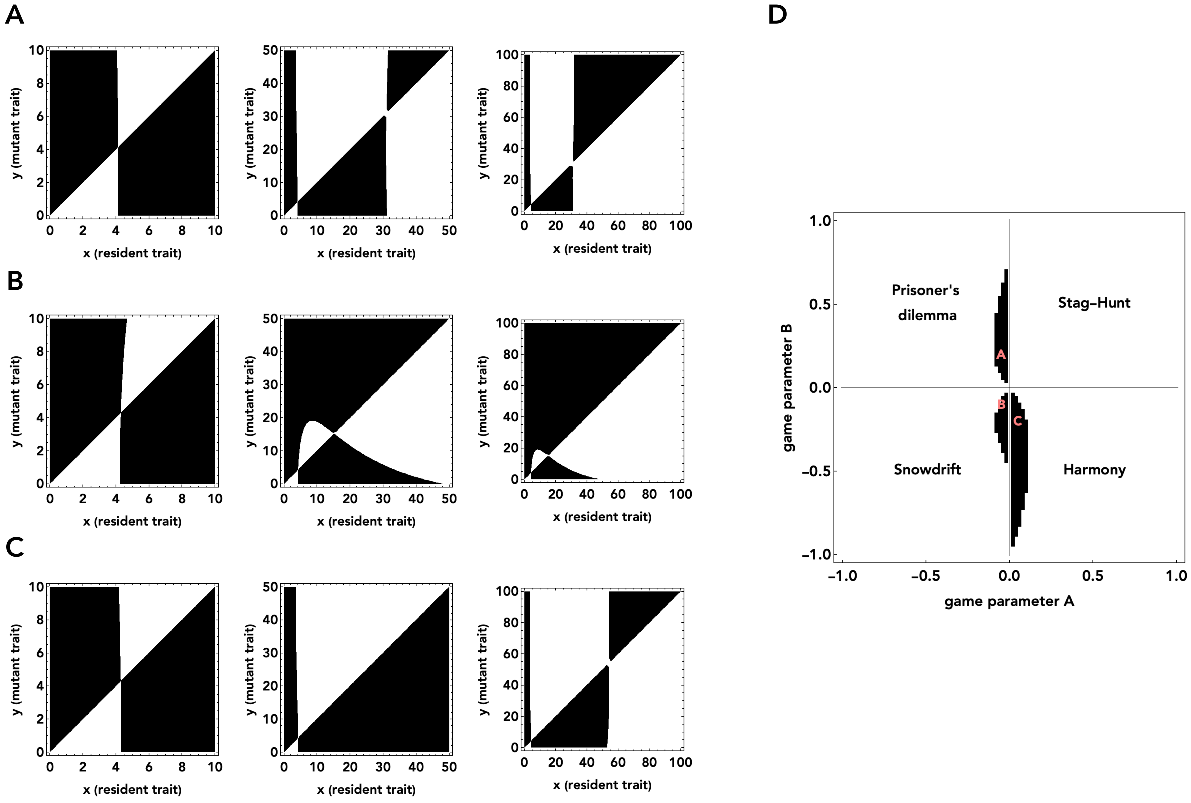

Fig. S5. A closer look at the weak dilemmas with a finite singular point shows the existence of a second singular point. Here, we display: **A,** a weak prisoner's dilemma ($A = -0.05$, $B = 0.2$), **B,** a weak snowdrift ($A = -0.05$, $B = -0.1$), and **C,** a weak harmony game ($A = 0.05$, $B = -0.2$). In each case, there is a unique singular point within our original range of payoff sensitivities, $\beta \in [0, 10]$. However, as we expand the range, we observe another singular point, above which payoff sensitivies are selectively favored to increase indefinitely. **D** We explore which games displayed in **Fig. 4** of the main text have the property that they give rise to more than one singular point. The respective sets of games exactly recovers the three additional areas in **Fig. 4**, where the payoff parameter A is close to being zero. In each case, the first singular point is convergence stable and the second is divergent. This indicates that above the second point, evolution happens in the direction of increasing payoff sensitivity (as it can be seen in the examples in panels **A**, **B** and **C**, also marked on top of panel **D** in pink).

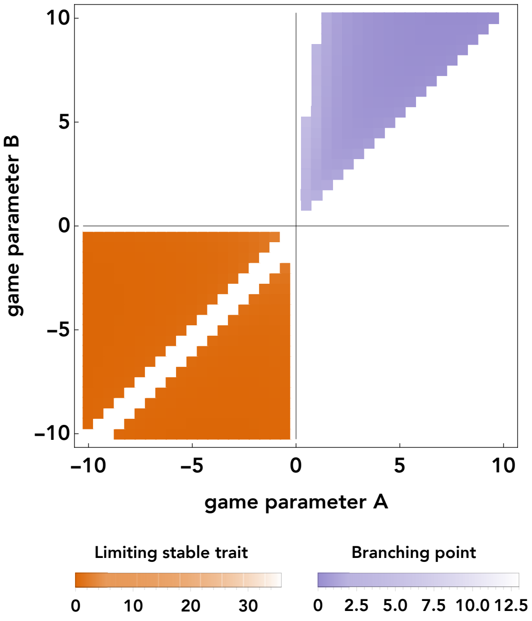

Fig. S6. Evolutionary outcomes across 2-strategy social dilemmas. We solve the equation $D(x^*) = 0$ for games in the shown range. When there is a solution x^* (colored), we derive its stability properties. In orange, the games that lead to a convergent and evolutionary stable trait. In purple, the games that lead to a convergent but unstable point, that is, a branching point. The color gradient represents the singular trait: the lighter, the higher the trait value is. When there is no solution to $D(x^*) = 0$, we verify that $D(x)$ is always positive (white). The game parameters A and B range between -10 and 10, in a 0.5 step.

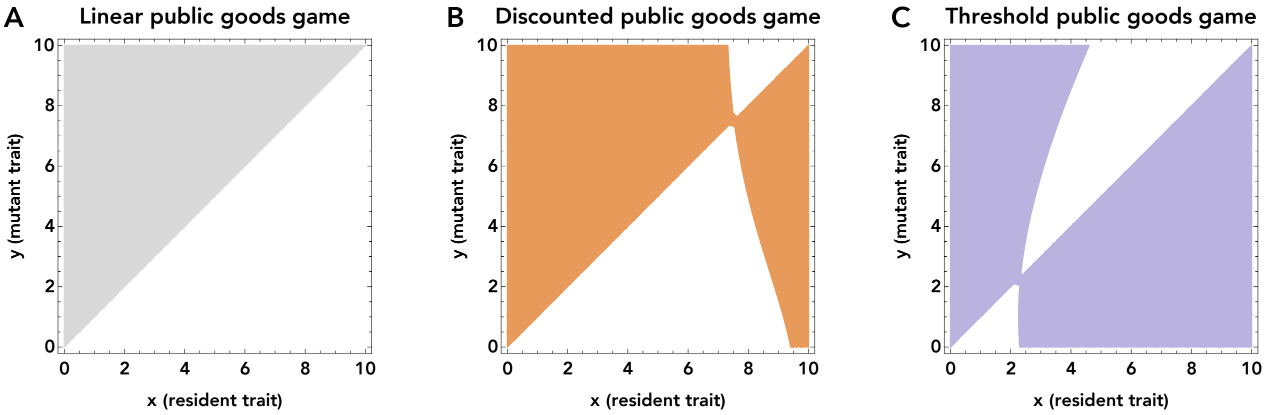

Fig. S7. Pairwise invasibility plots for multiplayer games. As before, the shaded areas represent a positive invasion fitness. **A,** Linear public goods game. The payoffs are defined by Eq. 27 with $N = 3$, $r = 2$, $c = 0.2$. **B,** Discounted public goods game. The payoffs are defined by Eq. 28 with $N = 3$, $r = 2$, $c = 0.2$, and $w = 0.5$. **C,** Threshold public goods game. The payoffs are defined by Eq. 29 with $N = 3$, $r = 2$, $c = 1$, and $M = 2$.

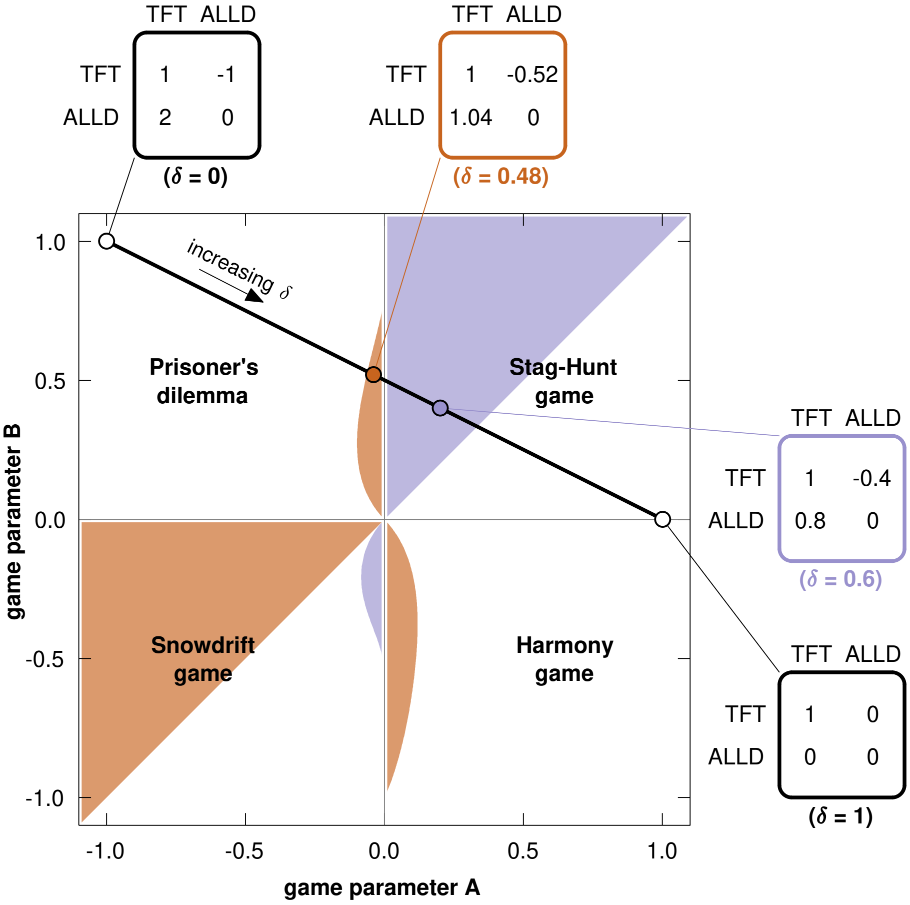

Fig. S8. Evolution of noisy learning in games of direct reciprocity. We provide an elementary analysis of the co-evolution of payoff sensitivity and direct reciprocity. To this end, we consider a repeated donation game where players can choose among two strategies, *ALLD* (Always defect) and *TFT* (Tit-for-Tat). The strategic nature of the game depends on the value of the discount factor δ. For small discount factors, the game between *ALLD* and *TFT* corresponds to a (one-shot) prisoner's dilemma. For larger discount factors, it becomes a stag-hunt game. Interestingly, this pairwise interaction can recover all dynamical regimes described previously (as in the analogous **Fig. 4** of the main text): payoff sensitivity may either evolve indefinitely, towards a finite value, or there may be branching. The black line depicts the possible games arising for different δ values, based on a repeated donation game with benefit $b=2$ and $c=1$.

B. Experience-weighted attraction learning.

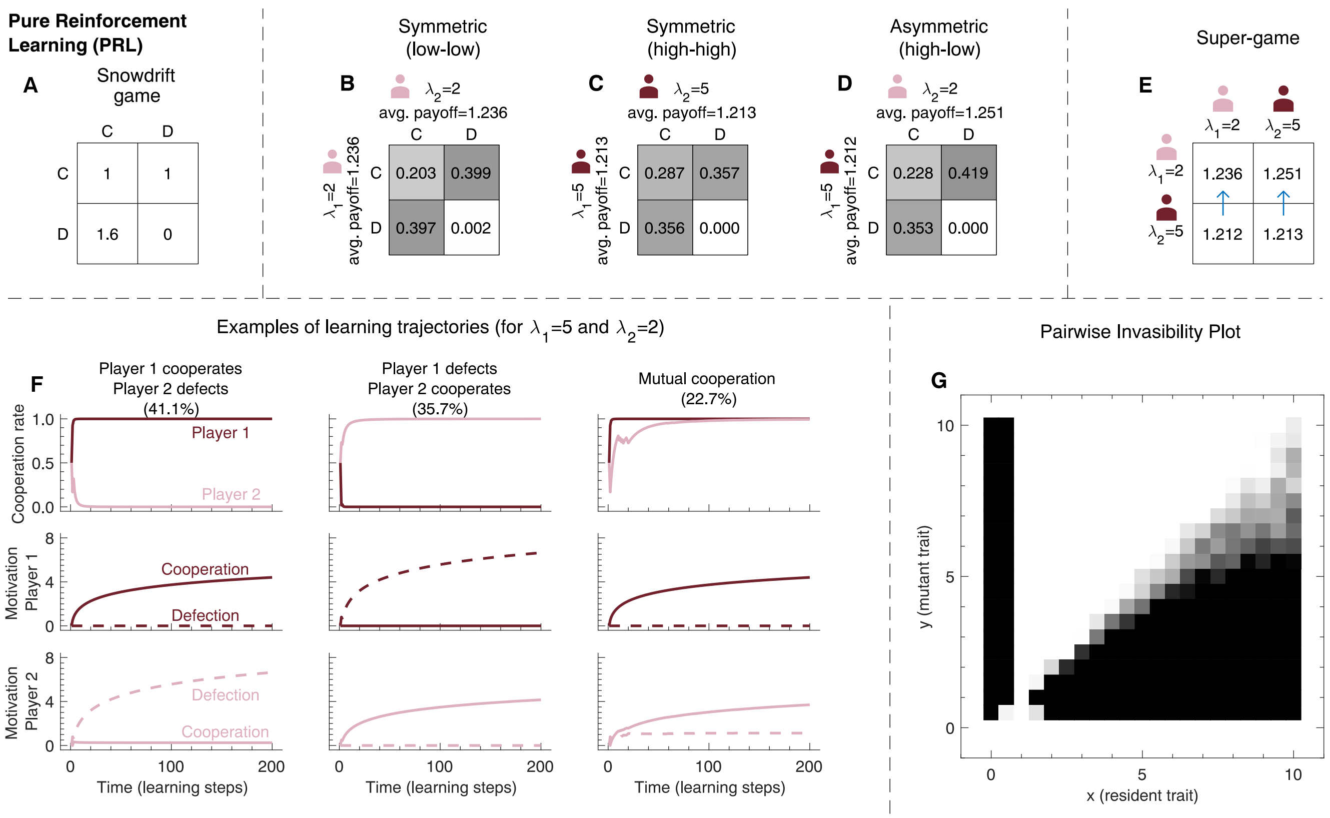

Fig. S9. Evolution of finite payoff sensitivity under pure reinforcement learning (PRL). A, As a first example of the possible dynamics under experience-weighted attraction, we consider a snowdrift game among two players who update their actions with pure reinforcement learning. **B–D,** Similar to our analysis of introspection dynamics, we compute expected payoffs when players can choose among two possible motivation sensitivities, here $\lambda \in \{2, 5\}$. However, while previously payoffs were calculated, here we estimate them through 10^5 simulations of the learning process. **E,** The respective super-game matrix suggests that $\lambda = 2$ dominates $\lambda = 5$, i.e., lower payoff sensitivies are favored. **F,** As we look at examples of the respective learning dynamics, most of the trajectories are in one of three classes: eventually either player 1 cooperates and player 2 defects (41.1% of another random sample of 1,000 cases), player 1 defects and player 2 cooperates (36.7% of cases), or both players cooperate (22.7%). To classify trajectories, we checked whether players cooperate at least 90% or at most 10% of the time. The unclassified trajectories are in neither of the three classes. **G,** As we perform similar simulations for a finite grid of λ values, we obtain a numerically computed pairwise invasibility plot. Here, a black color indicates that the mutant's numerical average payoff is at least 0.01 above the resident's. For payoff differences between 0 and 0.01, we use a grey shading, to also indicate the possible uncertainty given by sampling errors.

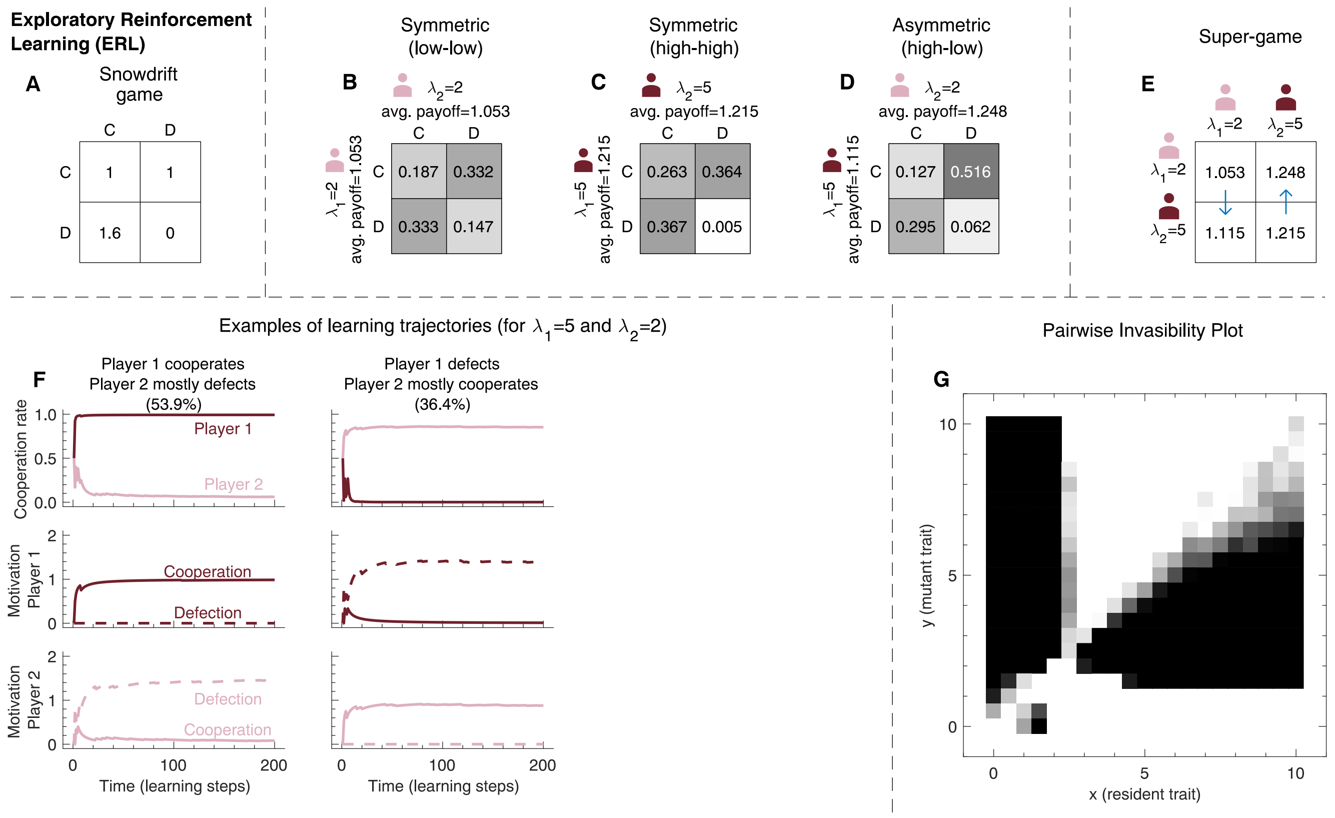

Fig. S10. Evolution of finite payoff sensitivity under exploratory reinforcement learning (ERL). A–D, The setup and the illustrated outcomes are similar to **Fig. S11**, but now for a different learning rule. **E,** In this case, the respective super-game is an anti-coordination game. **F,** Unlike in the previous figure, we no longer observe cases in which both players eventually cooperate with probability of at least 90%. Instead, we only observe cases in which one player mostly cooperates, whereas the other mostly defects. **G,** The pairwise invasibility plot again suggests the existence of a finite singular point.

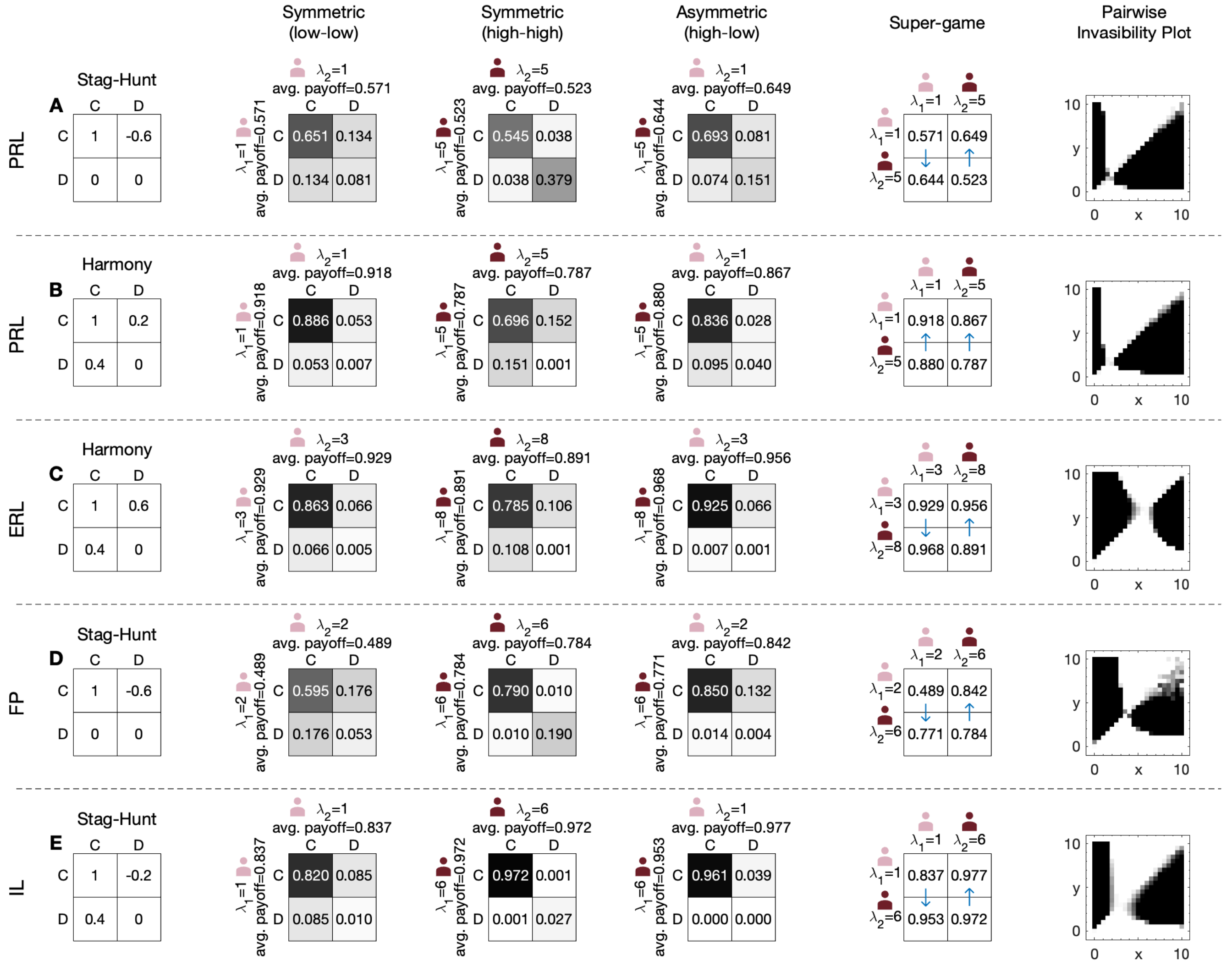

Fig. S11. Some further examples with a non-trivial adaptive dynamics. Again, we consider a similar setup as in Fig. S9 and **Fig.** S10, but this time using a broader range of games and learning rules. Examples are chosen such that the pairwise invasibility plot exhibits a finite singular point in each case. However, the respective mechanism responsible for the existence of that point is different across cases. In the stag-hunt games **A,D**, mixed groups (with a high λ and a low λ player) are more effective at coordinating at the optimal (C,C) outcome. In the harmony games **B,C**, in mixed groups the high λ player is more likely to cooperate, favoring the low λ player (even though this low λ player could further enhance its payoff by cooperating more often, too). Finally, in the stag-hunt game **E**, again in mixed groups the high λ player is most likely to cooperate (sometimes even if the low λ player does not).

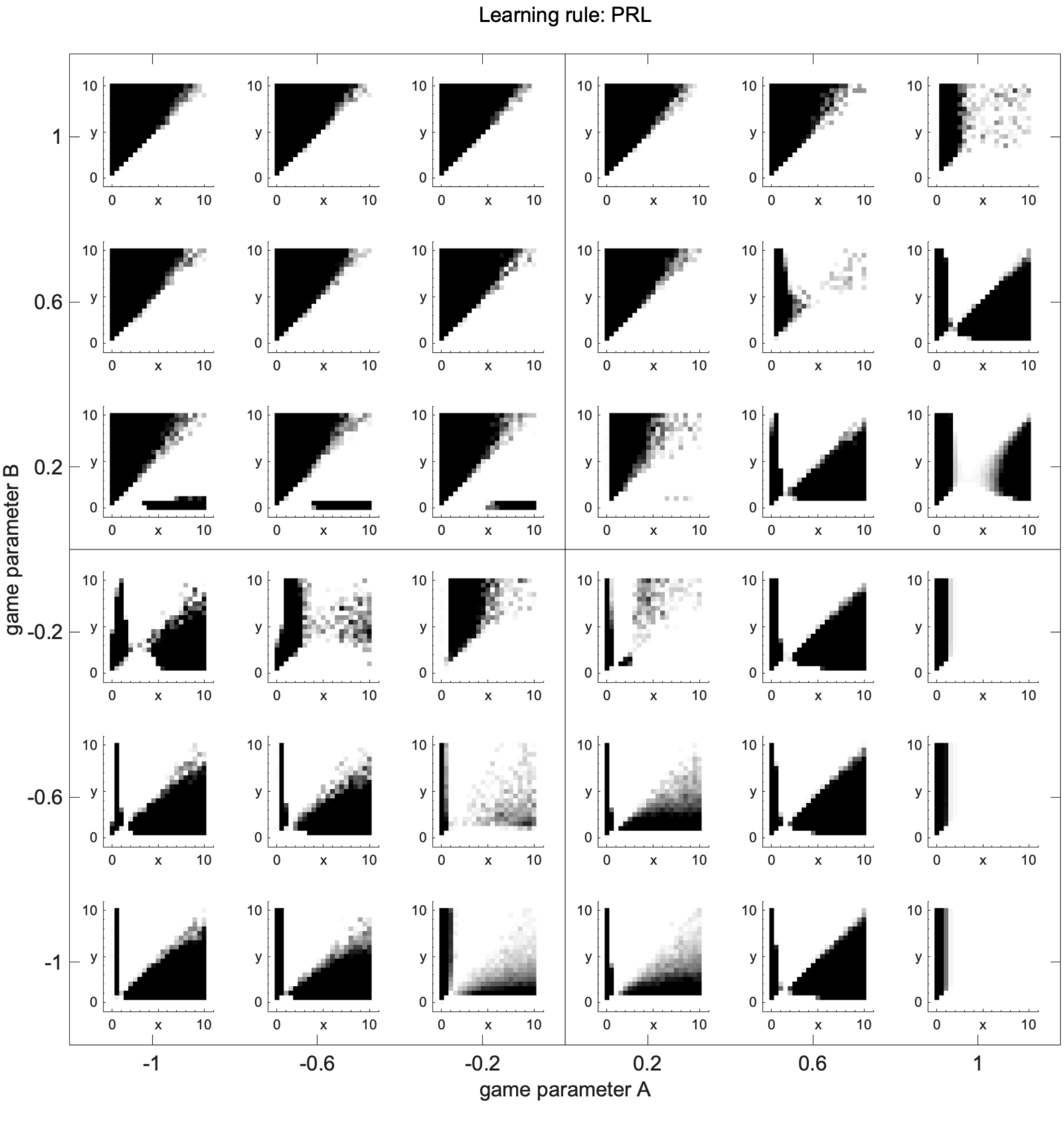

Fig. S12. Adaptive dynamics of payoff sensitivity under pure reinforcement learning (PRL). Similar to the previous figures, here we produce pairwise invasibility plots for a finite grid of different stage games. To this end, we systematically vary the payoff parameters $A, B \in \{-1.0, -0.6, -0.2, 0.2, 0.6, 1.0\}$. In each case, we systematically vary the players' motivation sensitivity $\lambda \in \{0, 0.5, 1.0, \ldots, 1\}$ and simulate the players' expected payoffs. We note that in the right-most column of pairwise invasibility plots (PIP), some harmony games yield PIPs that only have a black color for small resident motivation sensitivities. This means that either the respective payoff difference between mutant payoff and resident payoff is negative, or that it is very small. In those cases, adapting populations might evolve towards a motivation sensitivity that is merely sufficiently large, from which point onwards any further increases are selectively neutral.

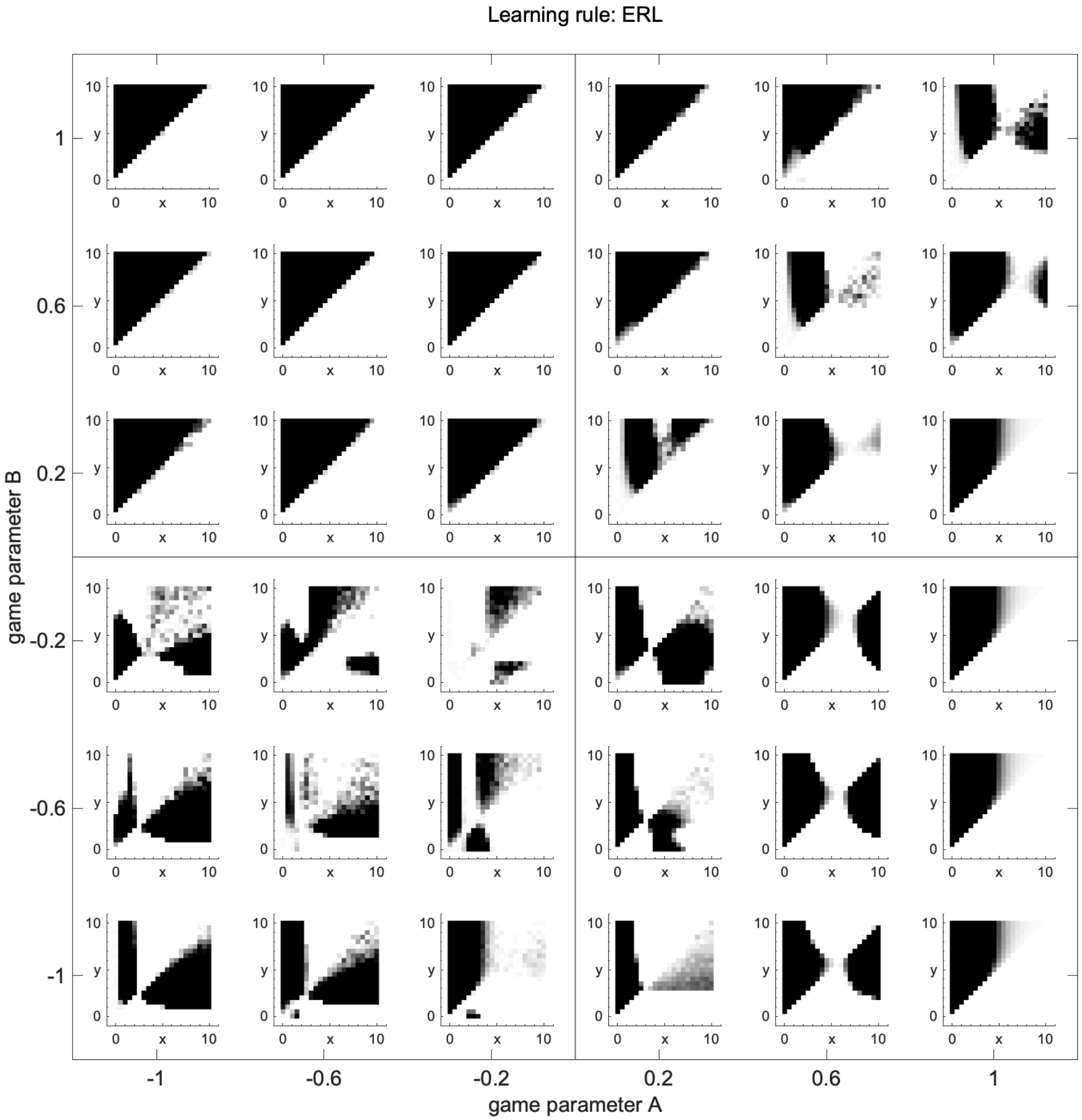

Fig. S13. Adaptive dynamics of payoff sensitivity under exploratory reinforcement learning (ERL). Similar to **Fig. S12**, but this time players update their strategies based on exploratory reinforcement learning instead of pure reinforcement learning.

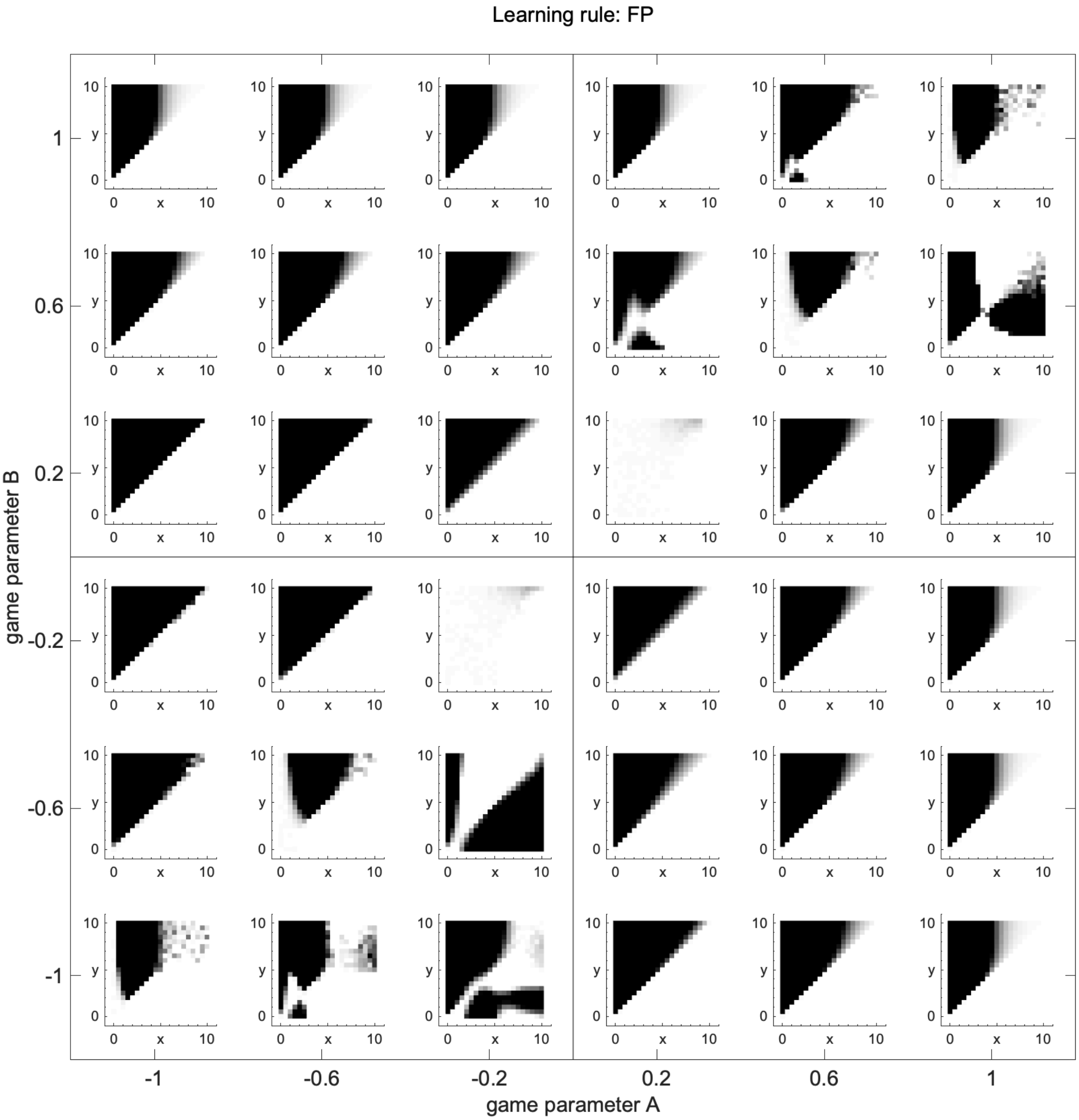

Fig. S14. Adaptive dynamics of payoff sensitivity under fictitious play learning (FP). Similar to the previous two figures, but this time players update their strategies based on fictitious play.

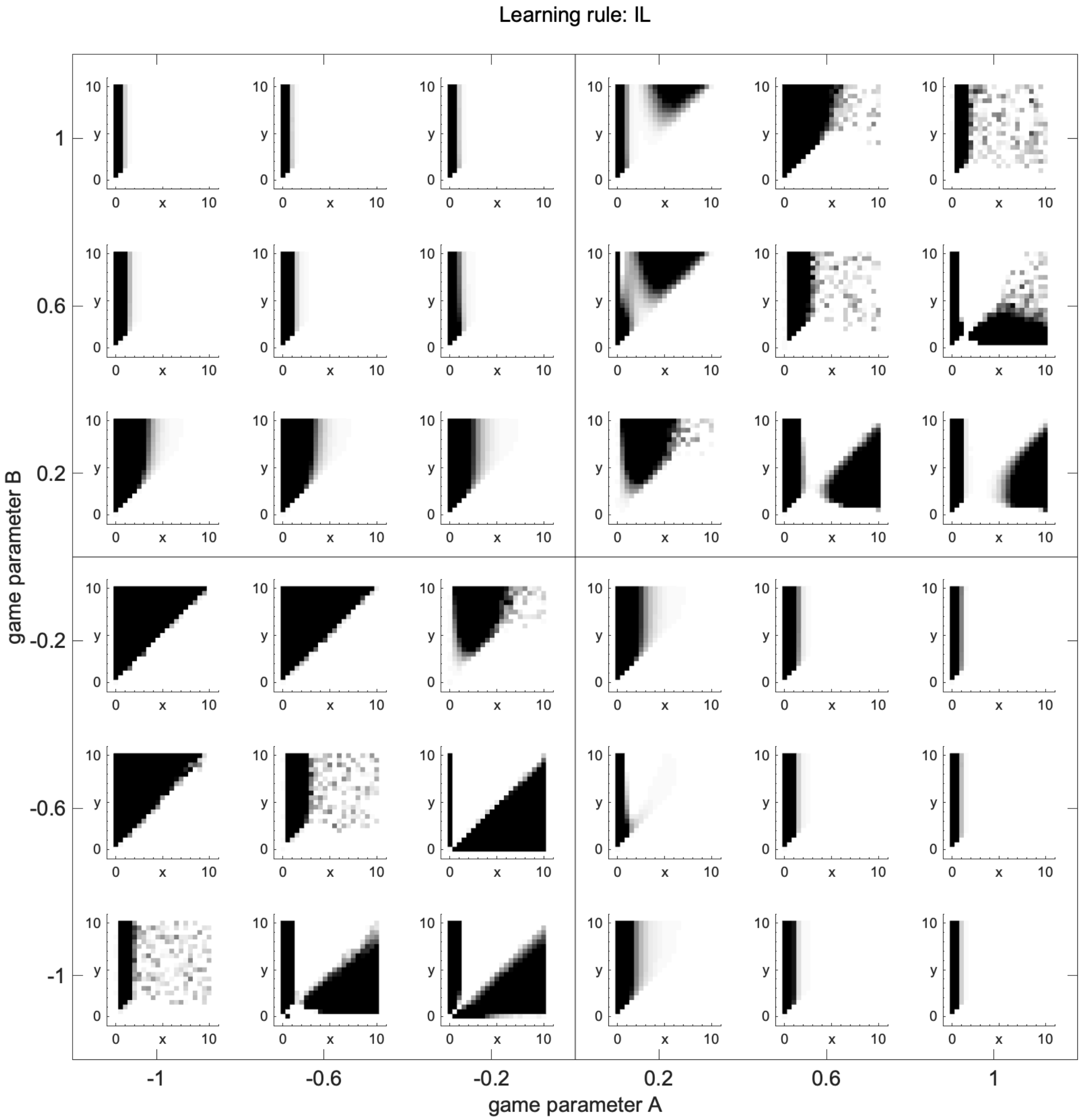

Fig. S15. Adaptive dynamics of payoff sensitivity under payoff-informed learning (IL). Similar to the previous three figures, but this time players update their strategies based on payoff-informed learning.

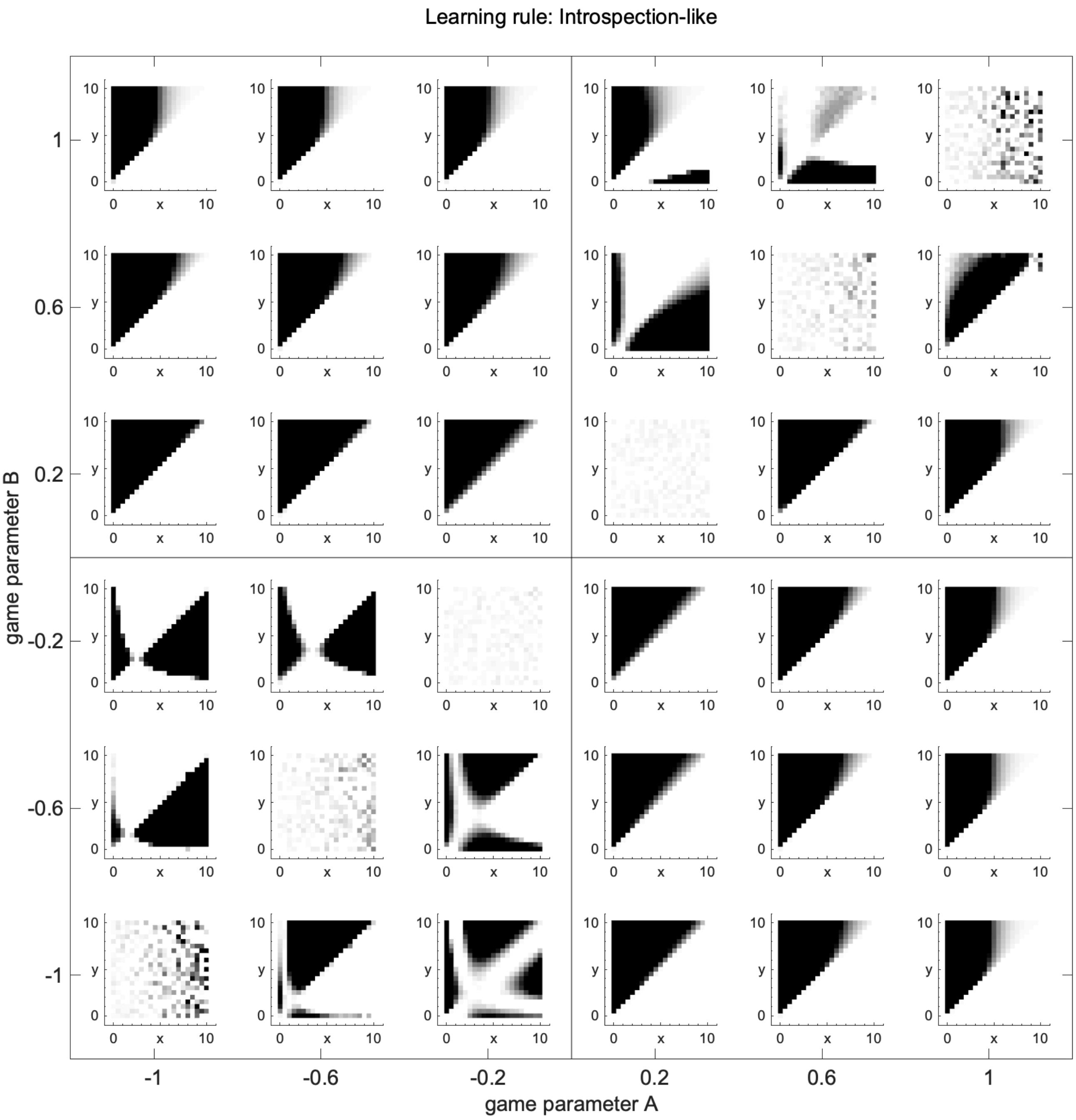

Fig. S16. Adaptive dynamics of payoff sensitivity under introspection-like learning. Similar to the previous figures, but this time players update their strategies based on introspection-like dynamics. Here, we only did 10^4 instead of 10^5 independent simulations to estimate payoffs, due to the better mixing properties of introspection-like dynamics, compared to some other dynamics of the EWA family. However, note the close agreement between this numerically generated figure and the analytically derived **Fig. S17**.

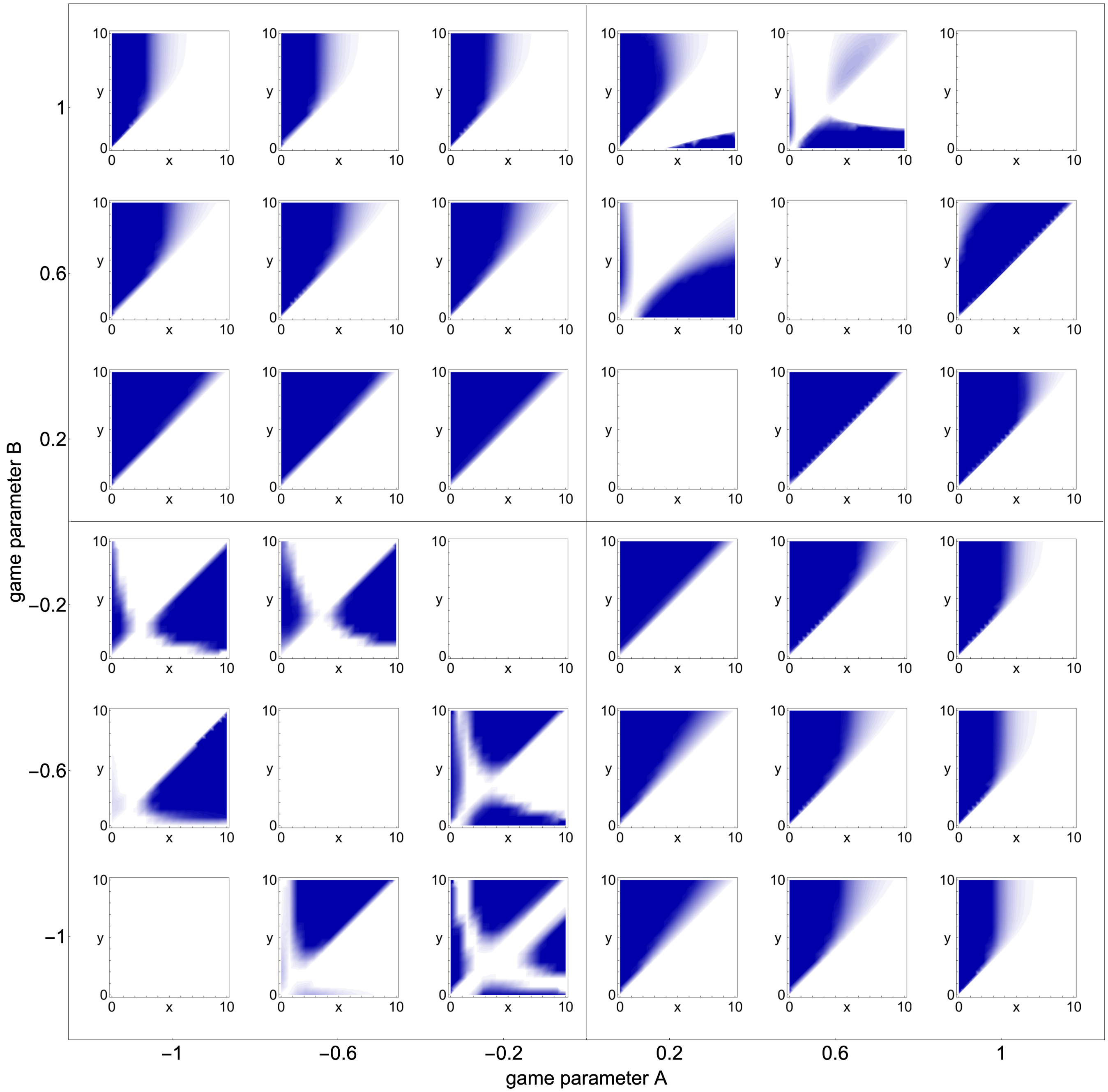

Fig. S17. Analytical results on the adaptive dynamics under introspection-like learning. This figure uses the same setup as in **Fig.** S16, but now regions are plotted according to the players' analytically derived payoffs. See **Section 3.**D for details.
